# Hypoxia-induced switch in SNAT2/SLC38A2 regulation generates endocrine resistance in breast cancer

**DOI:** 10.1073/pnas.1818521116

**Published:** 2019-05-31

**Authors:** Matteo Morotti, Esther Bridges, Alessandro Valli, Hani Choudhry, Helen Sheldon, Simon Wigfield, Nicki Gray, Christos E. Zois, Fiona Grimm, Dylan Jones, Eugene J. Teoh, Wei-Chen Cheng, Simon Lord, Dimitrios Anastasiou, Syed Haider, Alan McIntyre, Deborah C. I. Goberdhan, Francesca Buffa, Adrian L. Harris

**Affiliations:** ^a^Hypoxia and Angiogenesis Group, Cancer Research UK Molecular Oncology Laboratories, Weatherall Institute of Molecular Medicine, Department of Oncology, University of Oxford, Oxford OX3 9DS, United Kingdom;; ^b^Department of Biochemistry, Faculty of Science, King Abdulaziz University, Jeddah F6VM+J2, Saudi Arabia;; ^c^Computational Biology Research Group, Weatherall Institute of Molecular Medicine, John Radcliffe Hospital, University of Oxford, Oxford OX3 9DS, United Kingdom;; ^d^Cancer Metabolism Laboratory, Francis Crick Institute, London NW1 1ST, United Kingdom;; ^e^Computational Biology and Integrative Genomics, Department of Oncology, University of Oxford, Oxford OX3 7DQ, United Kingdom;; ^f^The Breast Cancer Now Toby Robins Research Centre, Division of Breast Cancer Research, The Institute of Cancer Research, London SW7 3RP, United Kingdom;; ^g^Cancer Biology, Division of Cancer and Stem Cells, The University of Nottingham, Nottingham NG7 2UH, United Kingdom;; ^h^Department of Physiology, Anatomy and Genetics, University of Oxford, Oxford OX1 3PT, United Kingdom

**Keywords:** amino acid transporter, breast cancer, cancer metabolism, ERα, hypoxia

## Abstract

Tumor hypoxia is associated with poor patient outcomes in estrogen receptor-α–positive (ERα^+^) breast cancer. Hypoxia is known to affect tumor growth by reprogramming metabolism and regulating amino acid (AA) uptake. Here, we show that the glutamine transporter, SNAT2, is the AA transporter most frequently induced by hypoxia in breast cancer, and is regulated by hypoxia both in vitro and in vivo in xenografts. SNAT2 induction in MCF7 cells was also regulated by ERα, but it became predominantly a hypoxia-inducible factor 1α (HIF-1α)–dependent gene under hypoxia. Relevant to this, binding sites for both HIF-1α and ERα overlap in SNAT2’s *cis*-regulatory elements. In addition, the down-regulation of SNAT2 by the ER antagonist fulvestrant was reverted in hypoxia. Overexpression of SNAT2 in vitro to recapitulate the levels induced by hypoxia caused enhanced growth, particularly after ERα inhibition, in hypoxia, or when glutamine levels were low. SNAT2 up-regulation in vivo caused complete resistance to antiestrogen and, partially, anti-VEGF therapies. Finally, high SNAT2 expression levels correlated with hypoxia profiles and worse outcome in patients given antiestrogen therapies. Our findings show a switch in the regulation of SNAT2 between ERα and HIF-1α, leading to endocrine resistance in hypoxia. Development of drugs targeting SNAT2 may be of value for a subset of hormone-resistant breast cancer.

## Significance

The hypoxic microenvironment in solid tumors is known to reduce the efficacy of anticancer treatments in many cancer types, including breast cancer. This study shows hypoxia induces an amino acid transporter, SNAT2, which then causes resistance to antihormone therapy. We show major interplay between genes induced by estrogen receptor and hypoxia. Hypoxia-inducible factor 1α compensates for the loss of expression of estrogen receptor-α (ERα) for maintaining SNAT2 expression under hypoxia or endocrine therapies. SNAT2 overexpression produces complete resistance to antiestrogen therapy in vivo and is induced in tamoxifen resistance, and its expression is associated with poor survival in breast cancer and resistance to endocrine therapy in ERα^+^ luminal B patients. Our findings thus have revealed a previously unidentified mechanism for antiestrogen resistance driven by tumor metabolism.

The estrogen receptor-α–positive (ERα^+^) subtype accounts for ∼70% of all newly diagnosed cases of breast cancer in Europe and the United States (1). The majority of these tumors depend on estrogen signaling, thereby providing the rationale for using antiestrogens as adjuvant therapy to treat breast cancer. Endocrine agents targeting ERα, such as tamoxifen, fulvestrant, or aromatase inhibitors, represent the cornerstone of systemic treatment of this breast cancer subtype (2). However, despite recent therapeutic advances, poor response and resistance limit the effectiveness of these agents in up to 30% of patients (3). Many mechanisms have been proposed to account for endocrine resistance such as genetic (e.g., loss of ERα expression, mutation, expression of truncated ER isoforms), activation by peptide growth factors, and epigenetic (e.g., methylation, promoter inhibition) changes within the tumor that activate hormone-independent mitogenic pathways (4). In addition to cancer cell-autonomous factors, the host microenvironment can contribute to endocrine resistance (5). Hypoxia is a key microenvironmental difference between tumor and normal tissues and is related to poor clinical prognosis and resistance to therapies in many solid tumors, including breast cancer (6). Indeed, hypoxia and increased expression of hypoxia-inducible factor 1α (HIF-1α), the key transcription factor mediating hypoxia response, are associated with endocrine resistance to neoadjuvant and adjuvant therapy in ERα^+^ breast cancers (7–9). We recently found that the *HIF-1α* gene bears a canonical ER-binding element that responds to estrogen signaling, demonstrating a direct regulatory link between the ERα and HIF-1α pathways in breast cancer (10). HIF-1α function could compensate for estrogen signaling when ERα function is compromised during hormone therapy, and thus cause resistance to tamoxifen or fulvestrant treatment (10).

A crucial mechanism by which the growth of cancer cells is promoted in hypoxic microenvironments is by metabolic reprogramming (11). The adaptation to hypoxia is coordinated by HIF-1α, which induces metabolic genes involved in increasing glycolytic flux (12). There is also up-regulation of glutamine metabolism to support proliferation, lipid biosynthesis, and protection from free radical stress (13). In addition to pyruvate derived from glycolysis, hypoxic cancer cells can supply substrates to the tricarboxylic acid (TCA) cycle to sustain mitochondrial adenosine 5′-triphosphate (ATP) production (anaplerosis) through the uptake of amino acids (AAs), such as glutamine, glycine, and serine. In particular, glutamine can fuel the TCA cycle through a series of biochemical reactions termed glutaminolysis (14).

This gives a strong rationale to identify hypoxia-induced metabolic alterations, particularly regarding glutaminolysis. Several studies have shown that endocrine therapy resistance in breast cancer cells is modulated by metabolic rewiring and tamoxifen-resistant cells are characterized by HIF-1α hyperactivation via modulation of Akt/mammalian target of rapamycin (mTOR), thus resulting in enhanced aerobic glycolysis and mitochondrial metabolism (15, 16). These data highlight the importance of metabolic adaptability of cancer cells for endocrine therapy resistance and suggest that targeting glutamine metabolism could be a novel approach to overcome resistance to endocrine therapies.

Because tumor cells under hypoxia have a high demand for AA, we hypothesized that AA transporters may be up-regulated selectively to meet this demand. Thus, we investigated the role of hypoxia-regulated AA transporters and in endocrine therapy resistance.

## Results

### Identification of Hypoxia-Induced AA Transporters by RNA-Sequencing.

To define the AA transporters that are involved in hypoxic adaptation, a panel of breast cancer cell lines was cultured in hypoxia (0.1% O_2_) for 24 h and RNA-sequencing (RNA-seq) was then performed. This panel included ER^+^ MCF7 and T47D; HER2^+^ SKBR3, BT474, and ZR751; and triple receptor-negative cell lines MDA-MB-231, MDA-MB-453, MDA-MB-468, BT20, and BT549. We analyzed transmembrane AA transporters expressed in tumors (17). As AAs are crucial for maintenance of the TCA cycle throughout anaplerosis, we applied in silico hierarchical clustering and supervised exploratory analysis to evaluate which AA transporters promoting the uptake of specific substrates in the TCA cycle might be crucial for maintaining anaplerotic pathways in hypoxia. We assessed the substrates of these AA transporters: glucogenic versus ketogenic AAs, essential versus nonessential AAs, and the entry of their substrates into the TCA cycle.

**Fig. 1.**
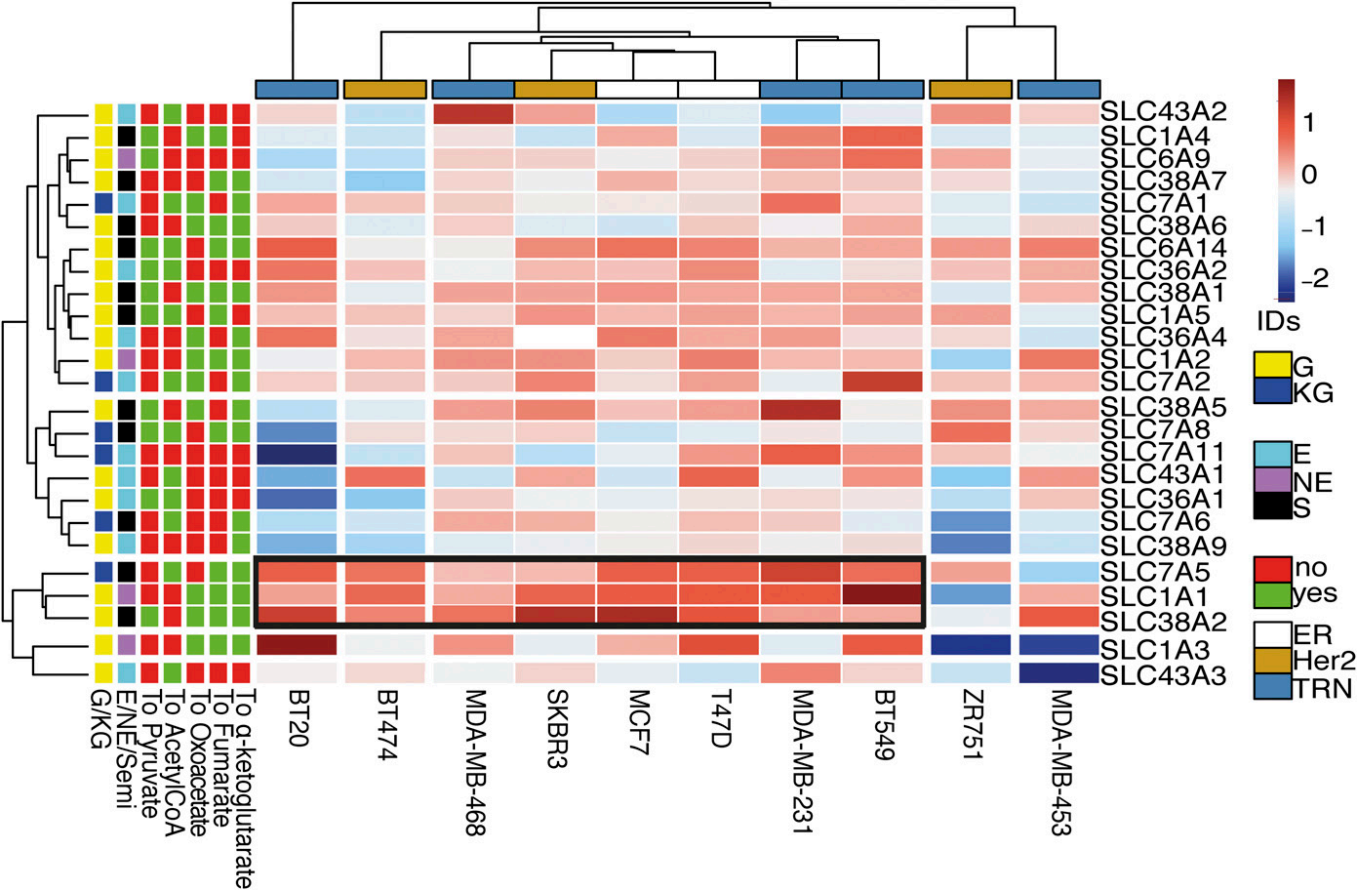
SLC38A2 (SNAT2), SLC7A5, and SLC1A1 are the hypoxic-induced AA transporters in breast cancer cell lines. Ten breast cancer cell lines (columns) cultured in normoxia and hypoxia and submitted to RNA-seq were arranged by supervised average-linkage hierarchical clustering. Heatmap colors represent relative mRNA expression of the selected AA transporters (rows), with higher (red) or lower (blue) expression in hypoxia compared with normoxia. AA transporters that mediate the uptake of gluconeogenic (G) or ketogenic (KG) essential (E) or nonessential (NE) AAs that enter specific steps of the TCA cycle (α-ketoglutarate, fumarate, oxaloacetate, and pyruvate) were clustered. The black box represents the cluster composed of three AA transporters (*SLC1A1*, *SLC7A5*, and *SLC38A2*) in eight cell lines. S, semiessential; TRN, triple receptor-negative.

Several AA transporters are up-regulated under hypoxia in breast cancer cell lines (Dataset S1), but only three transporters among the 25 investigated are consistently up-regulated and clustered under hypoxia in more than three cell lines. *SLC1A1*, *SLC7A5* (*LAT1*), and *SLC38A2* (*SNAT2*) transporters were coregulated in MCF7, T47D, SKBR3, MDA-MB-231, MDA-MB-468, BT549, BT20, and BT474 (Fig. 1).

The branched-chained AA transporter *LAT1* (*SLC7A5*) is a HIF-2α target (18), while *SLC1A1* is a glutamate HIF-dependent transporter (19) (Fig. 1). The third AA transporter in the cluster strongly up-regulated across a wide spectrum of cell lines under hypoxia is SNAT2, which is involved in neutral AA intake (20). Theoretically, the AAs provided by these three transporters could account for all AA precursors of TCA cycle metabolites (*SI Appendix*, Fig. S1*A*). SLC7A5 can support the uptake of ketogenic AAs, SLC1A1 the uptake of glutamate, and SNAT2 the uptake of other neutral gluconeogenic AAs such as glutamine, alanine, glycine, and serine. Validation of induction by hypoxia by qPCR assay of these AA transporters was performed in six different cell lines (*SI Appendix*, Fig. S1*B*). Further analysis of RNA-seq data of four breast cancer cell lines with different receptor phenotypes (MCF-7, SKBR3, MDA-MB-231, and MDA-MB-468) demonstrated that, in addition to the full-length *SNAT2* transcript (isoform 1), there were truncated splice variants of *SNAT2* (isoforms 2 and 3) encoding shorter proteins lacking three and six transmembrane domains (*SI Appendix*, Fig. S1*C*). The three isoforms encode for proteins (predicted by TMHMM Server) of 56, 45, and 38 kDa, respectively (*SI Appendix*, Fig. S1*D*). A qPCR assay for specific amplification of only the two main isoforms (1, 2) was used, as isoform 3 was not detected. In the cell lines where SNAT2 was induced, there was an increase of expression of both *SNAT2* messenger RNA (mRNA) isoforms 1 and 2 after 24 h of 0.1% O_2_ (*SI Appendix*, Fig. S1*E*).

### SNAT2 Protein Is Up-Regulated Under Hypoxia In Vitro Mainly in a HIF-1α–Dependent Manner.

**Fig. 2.**
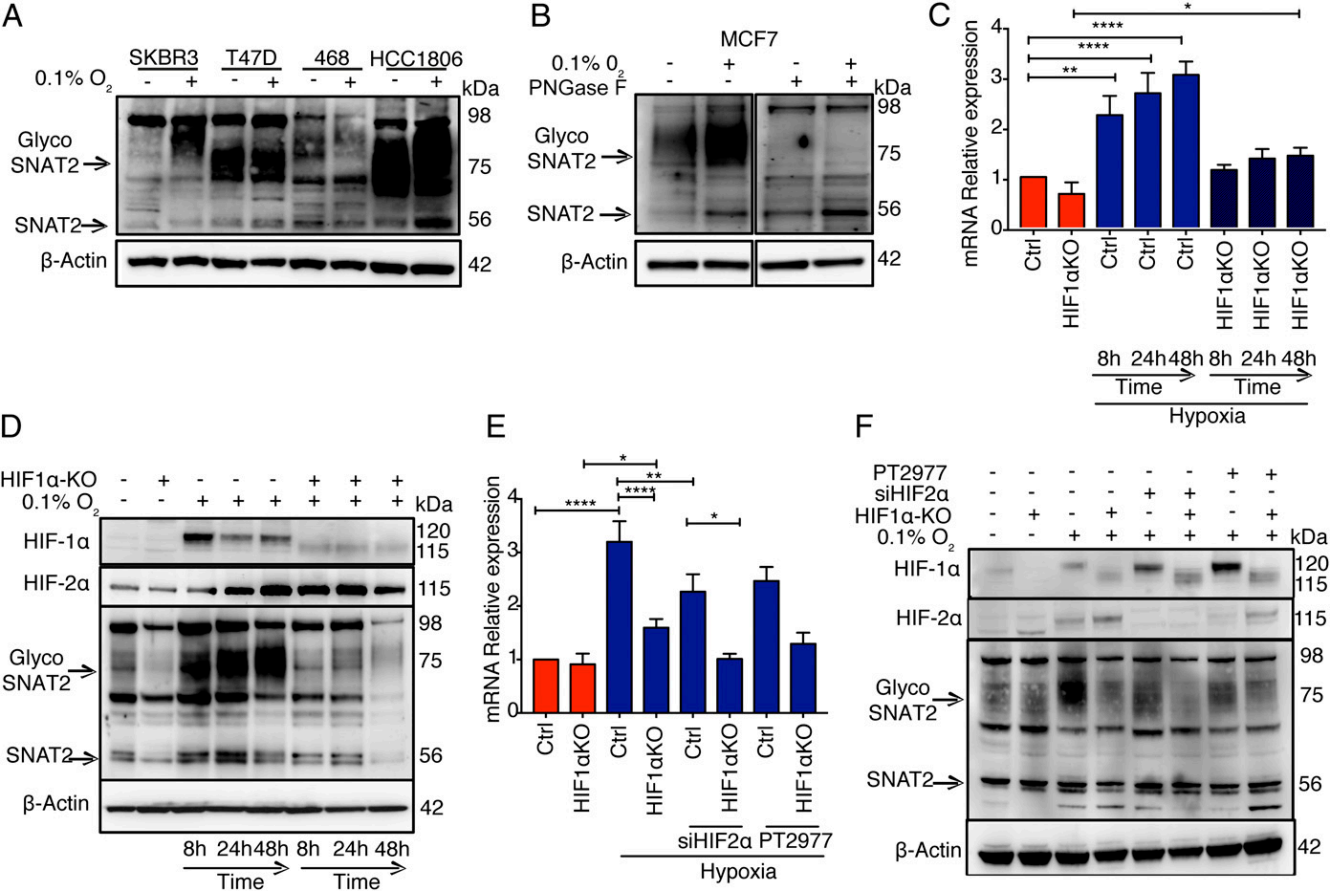
SNAT2 is a hypoxia-induced gene and is mainly regulated by HIF-1α. (*A*) Immunoblot of four breast cancer cell lines from different histotypes showing the heterogeneity in basal SNAT2 expression and their response to 48 h of hypoxia. (*B*) *N*-linked deglycosylation assay using PNGase in MCF7 cell lysate in normoxia and hypoxia. (*C*) MCF7 parental and MCF7-HIF1α knockout cells (^−/−^) were cultured in normoxia (red) or in hypoxia (blue, 0.1% O_2_) for 8, 24, and 48 h. mRNAs were analyzed by RT-qPCR and normalized to 21% O_2_. *SNAT2* mRNA was normalized to the mean of *β-actin* and *RPL11*. Ctrl, control; KO, knockout. (*D*) Immunoblot validation in the same experiment described above. β-Actin is shown as a loading control. (*E*) MCF7 parental and MCF7-HIF1α (^−/−^) cells were transfected with scrambled control or siRNAs against *HIF-2α* (si*HIF2α*) or treated with PT2779 (HIF-2α inhibitor, 1 μM) and cultured in normoxia (red) or in hypoxia (blue, 0.1% O_2_) for 48 h. mRNAs were analyzed by RT-qPCR. *SNAT2* mRNA was normalized to the mean of *β-actin* and *RPL11*. (*F*) Immunoblot of SNAT2, HIF-1α, and HIF-2α in the same experiment described above (*E*). β-Actin is shown as a loading control. Error bars indicate SD. *P < 0.05 vs. 21% O_2_, **P < 0.01 vs. 21% O_2_, ****P < 0.0001 vs. 21% O_2_; one-way ANOVA (*n* = 3 for all experiments).

We examined SNAT2 protein expression (Fig. 2*A*) using an antibody against SNAT2 isoform 1. Basal RNA (*SI Appendix*, Fig. S2*A*) and protein and hypoxic *SNAT2* induction varied among cell lines. Measurement of *SNAT2* mRNA levels in normoxia and hypoxia in a number of additional human cancer cell lines derived from different tissues showed that *SNAT2* expression is widely up-regulated after 48 h of hypoxia in many cancer cell types (*SI Appendix*, Fig. S2*B*).

MCF7 ERα^+^ cells were used for further investigation. To determine whether the 75- to 90-kDa bands represented a glycosylated isoform of the 56-kDa SNAT2 isoform 1 protein, samples were deglycosylated with an *N*-linked glycosidase [peptide:*N*-glycosidase F (PNGase F)]. The disappearance of the 75- to 90-kDa bands after PNGase F treatment indicates that these bands represent the *N*-glycosylated form of the molecule (Fig. 2*B*).

The effect of *HIF-1α* or *HIF-2α* depletion by small interfering RNA (siRNA) during this period of hypoxia (*SI Appendix*, Fig. S2 *C* and *D*) was studied. Hypoxic induction of *SNAT2* mRNA and protein expression was significantly reduced by *HIF-1α* siRNA (*P* < 0.01, *n* = 3) (*SI Appendix*, Fig. S2*C*). The siRNA knockdown was validated by immunoblots of the expression of both HIF-1α and HIF-2α, and by measuring the carbonic anhydrase 9 (CA9) protein levels (HIF-1α–dependent gene) (21) (*SI Appendix*, Fig. S2*D*).

To further assess the role of HIF-1α on SNAT2 induction, we used an MCF7-HIF1α (^−/−^) cell line generated through clustered regularly interspaced short palindromic repeats (CRISPR)/Cas9 methodology. The time course of hypoxic induction of SNAT2 in MCF7 cells by qPCR and Western blotting peaked at 48 h. The hypoxic induction of SNAT2 mRNA and protein was significantly reduced in the MCF7-HIF1α (^−/−^) (*P* < 0.01, *n* = 3) after 48 h of hypoxia (0.1% O_2_) (Fig. 2 *C* and *D*). The SNAT2 mRNA was reduced by around 2.2-fold at 48 h (*P* < 0.001; Fig. 2*C*), while the glycosylated SNAT2 protein was reduced by 3.6-fold at 48 h of hypoxia (*SI Appendix*, Fig. S2*E*). There was no compensatory hyperinduction of HIF-2α, apart from an earlier peak in MCF7-HIF1α (^−/−^), which was induced to similar levels in both cell lines (Fig. 2*D*).

Interestingly, HIF-2α depletion by siRNA or by a specific inhibitor (PT2977, 1 μM) reduced both SNAT2 mRNA and protein hypoxic induction (0.1% O_2_) (*P* = 0.004) (Fig. 2 *E* and *F*) in the control cells. The HIF-2α depletion (siRNA or inhibitor) in MCF7-HIF1α (^−/−^) reduced the *SNAT2* mRNA induction to the basal levels (although this was not statistically significant) (Fig. 2 *E* and *F*). These data suggest that HIF-1α is responsible for around 65% of mRNA hypoxic induction of *SNAT2* and HIF-2α is responsible for the remaining 35% of induction, but that it does not compensate for the effect of HIF-1α.

We then stably overexpressed HIF-1α by retroviral vector-mediated transduction in MCF7 (MCF7-HIF1α-o). Measurement of *SNAT2* and *CA9* mRNA levels in normoxia showed increased SNAT2 and CA9 expression in MCF7-HIF1α-o, while no further increase was seen in hypoxia (*SI Appendix*, Fig. S2*E*).

### SNAT2 Is Up-Regulated under Hypoxia In Vivo.

**Fig. 3.**
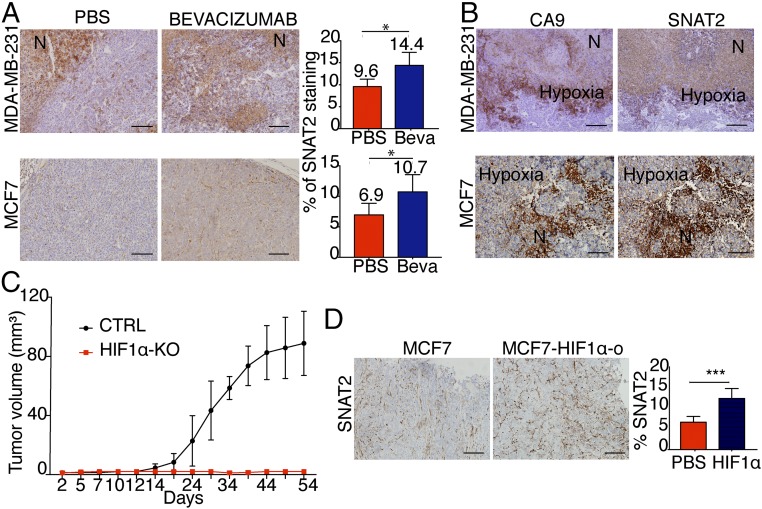
SNAT2 is up-regulated in vivo by hypoxia. (*A*) Representative SNAT2 immunostaining in MDA-MB-231 (first row) and MCF7 (second row) xenografts treated with phosphate-buffered saline (PBS; *n* = 5) or bevacizumab (Beva; *n* = 5). (Scale bars: 100 μm.) SNAT2 protein expression quantification by ImageJ in the same xenografts is shown. N, necrosis. (*B*) Representative SNAT2 and CA9 (hypoxia) immunostaining in MDA-MB-231 and MCF7 xenografts treated with bevacizumab, showing colocalization of SNAT2 in hypoxic areas of the tumors. (Scale bars: 100 μm). (*C*) Xenograft growth curves of MCF7 parental (*n* = 6) and MCF7-HIF1α KO cells (*n* = 7). CRTL, control. (*D*) Representative immunohistochemical images of SNAT2 staining in MCF7 parental and MCF7-HIF1α-o xenografts and a bar chart of scoring. (Scale bars: 100 μm.) Error bars indicate SD. *P < 0.05, ***P < 0.01; nonparametric Mann–Whitney test (*n* = 5 per group).

To determine whether hypoxic induction of SNAT2 occurs also in solid tumors, two breast cancer cell lines, MCF-7 and MDA-MB-231, were injected into nude mice and grown as xenografts. MDA-231 cells were used to assess the effect of hypoxia independent of ER expression. The mice were treated with either saline control or the VEGF inhibitor, bevacizumab, which slowed tumor growth temporarily (*SI Appendix*, Fig. S3 *A* and *B*). Treatment with bevacizumab increased SNAT2 protein levels in both MCF7 and MDA-MB-231 xenografts (*P* < 0.05; Fig. 3*A*). Human *SNAT2* mRNA and *CA9* mRNA were up-regulated also (Fig. 3 *A* and *B*). Bevacizumab decreased the percentage of blood vessels (CD31), and, accordingly, the proportion of necrosis (hematoxylin/eosin) and the percentage of CA9-positive cells (hypoxia) were greater in bevacizumab-treated xenografts compared with phosphate-buffered saline–treated xenografts (*SI Appendix*, Fig. S3 *E* and *F*).

Furthermore, we found that SNAT2 colocalized in both xenografts in the same areas where CA9 and pimonidazole were expressed (hypoxic areas) (Fig. 3*B* and *SI Appendix*, Fig. S3 *E* and *F*). To further investigate the effect of HIF-1α on SNAT2 expression in vivo, an orthotopic xenograft tumor was established using MCF7-HIF1α (^−/−^) and MCF7-HIF1α-o cells. MCF7-HIF1α (^−/−^) showed no tumor growth in nonobese diabetic severe combined immunodeficient (SCID)-γ (NSG) mice (Fig. 3*C*), indicating that HIF-2α did not compensate for the absence of HIF-1α.

HIF-1α overexpression resulted in increased SNAT2 expression (Fig. 3*D*).

These data show that SNAT2 is regulated by hypoxia in vivo, as well as in vitro.

### ERα and HIF-1α Bind to Overlapping Sites in the SNAT2 Promoter in MCF7 Cells but Do Not Act Synergistically.

**Fig. 4.**
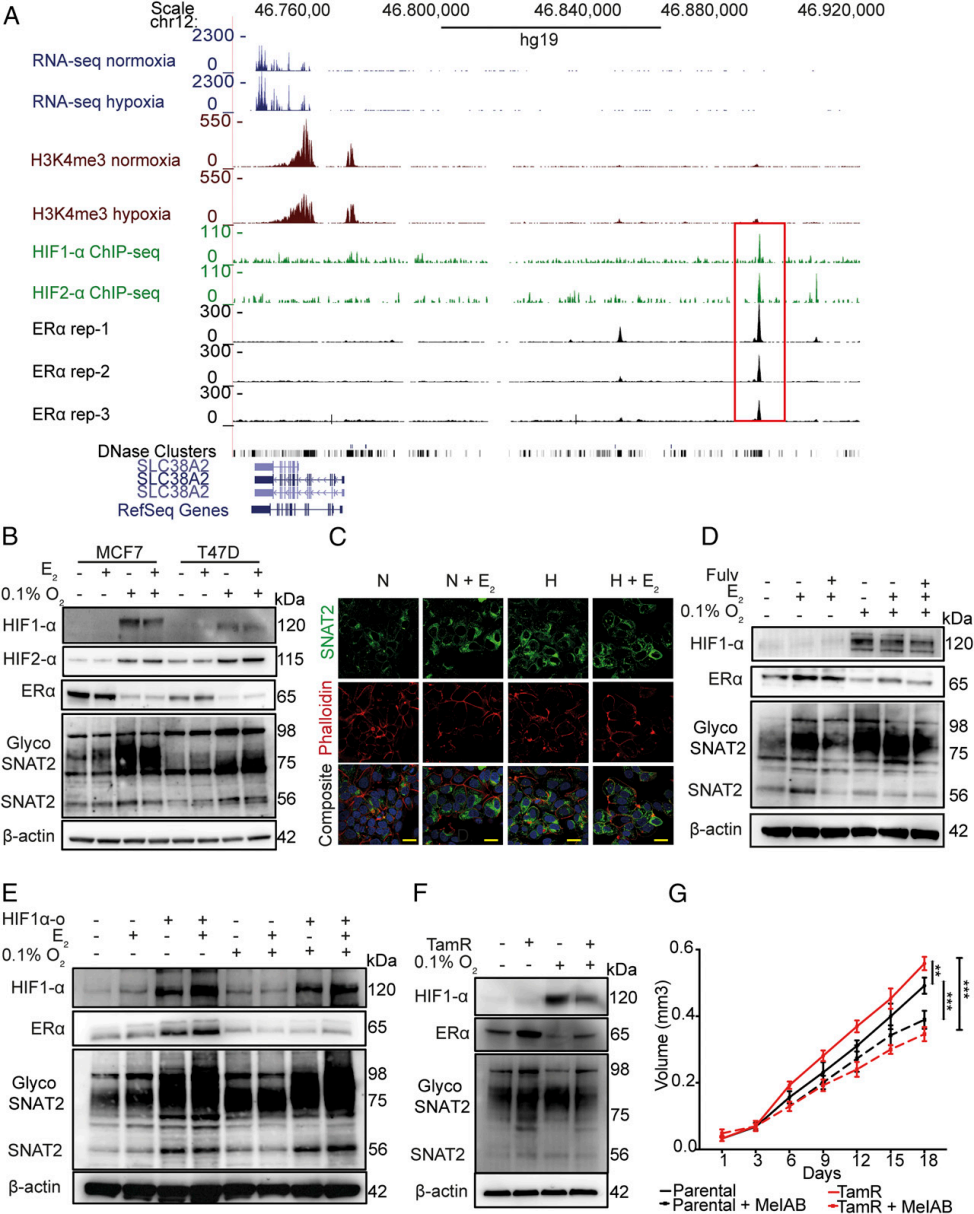
SNAT2 expression is independently modulated by ERα and HIF-1α, and is up-regulated in a tamoxifen-resistant cell line. (*A*) RNA-seq and ChIP-seq genome-browser tracks illustrating occupancy of *HIF-1α* (*Top*), *HIF-2α* (*Middle*), and *ERα* (*Bottom*) at the same genomic coordinates on chromosome 12. The same plots for H3K4me3 are shown. Peaks (red box) represent the areas where transcription factors interact with DNA. (*B*) Western blotting for SNAT2, ERα, HIF-1α, and HIF-2 in MCF-7 and T47D cells treated with 10 nM E_2_ in normoxia and hypoxia (0.1% O_2_) for 48 h. β-Actin is shown as a loading control. (*C*) Confocal microscopy of SNAT2 (green), phalloidin (F-actin, red), and DAPI (blue) in MCF7 in normoxia (N) and hypoxia (H; 0.1% O_2_) with or without E_2_ treatment (E; 10 nM) for 24 h. (*D*) MCF7 cells were grown in a charcoal-stripped, phenol-free medium for 3 d and then incubated with or without fulvestrant (Fulv; 10 μM) and with or without 10 nM E_2_ in normoxia and hypoxia (0.1% O_2_) for 48 h. β-Actin is shown as a loading control. (*E*) MCF7 control and MCF7-HIF1_α-o_ cells were treated with or without 10 nM E_2_ in normoxia and hypoxia (0.1% O_2_) for 48 h. β-Actin is shown as a loading control. (*F*) Representative Western blots of parental (MCF7-par) and MCF7 tamoxifen-resistant cells (MCF7-TamR) cultured in normoxia and hypoxia (0.1% O_2_) for 48 h. (*G*) Graph of the effect of MeIAB (10 mM) treatment on MCF7 and MCF7-TamR spheroid growth. Error bars indicate SD. **P < 0.05, ***P < 0.01; two-way ANOVA (*n* = 4).

Previous studies showed that the expression of SNAT2 was increased in ER^+^ breast cancer cell lines after 17β-estradiol (E_2_) stimulation, and an estrogen response element was described in the SNAT2 promoter in rat mammary glands during gestation (22, 23). Because SNAT2 was among the 202 genes bound by HIF-1α and ERα (10), we investigated the regulation of SNAT2 expression by estrogen and hypoxia. Utilizing RNA-seq and HIF-1α and HIF-2α chromatin immunoprecipitation (ChIP)-seq data in MCF-7 cells, we confirmed the presence of HIF-binding site sequences (RCGTG) for both HIF-1α and HIF-2α upstream of the promoter region of SNAT2 (24) (Fig. 4*A*). We aligned the ChIP results of HIF-1α in MCF7 with previous publicly available ERα-seq ChIP-seq data from the Encyclopedia of DNA Elements (ENCODE) in the same cell line (25). Both binding sites for HIF-1α and ERα are overlapping in the genome in *cis*-regulatory elements, suggesting a potential interaction between these two transcription factors for SNAT2 induction (Fig. 4*A* and *SI Appendix*, Fig. S4*A*). Interestingly SNAT1, another system A transporter, showed ERα-binding sites but not HIF-binding sites (*SI Appendix*, Fig. S4*A*).

We decided to investigate if an interaction existed between HIF-1α and ERα in driving SNAT2 expression. We cultured MCF7 and T47D cells in normoxia or hypoxia and treated with or without E_2_. Estrogen supplementation induced SNAT2 expression in normoxia. Small additive effects were seen in hypoxia in MCF7, but levels of ERα protein significantly decreased (Fig. 4*B*). A similar induction of SNAT2 under estradiol and hypoxia, but with a different magnitude, was also seen for T47D cell line.

To evaluate if estradiol and hypoxia might be responsible for different localization of SNAT2, we examined SNAT2 distribution using immunofluorescence after hypoxia (0.1% O_2_) and estradiol treatment (10 nM). Hypoxia and estradiol resulted in a significant increase in SNAT2 staining intensity, but none of the treatments affected its distribution (green) (Fig. 4*A* and *SI Appendix*, Fig. S4*C*). SNAT2 was located on punctate structures and the plasma membrane (F-actin, red), but the majority was on an intracellular compartment, which has been reported to be the trans-Golgi network (TGN) (26) (Fig. 4*C*). Time course experiments of estradiol supplementation in MCF7 in normoxia and hypoxia confirmed the estradiol dependency in normoxia with no significant additive effects in hypoxia (*SI Appendix*, Fig. S4*D*).

### ERα and HIF-1α Signaling Regulate SNAT2 Expression Independently.

To further confirm SNAT2 as an ERα- and HIF-1α–dependent gene, we cultured MCF7 cells for 5 d in charcoal-stripped, phenol-free medium and treated them with E_2_ with or without fulvestrant (ICI182780) in normoxia and hypoxia. Fulvestrant treatment abolished estradiol-dependent SNAT2 induction in normoxia, but only a small effect was seen in hypoxia (Fig. 4*E*), suggesting a major role for HIF-1α in this condition.

We then treated the MCF7-HIF1α-o cells with or without estradiol in normoxia and hypoxia. SNAT2 was induced in normoxia and hypoxia in MCF7-HIF1α-o cells. When wild-type cells were supplemented with E_2,_ an increase of SNAT2 was seen only in normoxia but not in hypoxia (Fig. 4*E*), while in MCF7-HIF1α-o cells, an increased level of SNAT2 was seen in both conditions, suggesting that HIF1-o might enhance ER signaling as previously reported (10) (Fig. 4*E*).

We then evaluated the 202 genes that we have previously shown to have binding sites for both HIF-1α and ERα (10). Using different publicly available experiments (24, 25), we confirmed 179 genes with both HIF-1α– and ERα-binding sites. Only 31 of these genes (31 of 179, 17.3%) had HIF-1α– and ERα-binding sites located in the same genomic region (*SI Appendix*, Table S1). By measuring mRNA, we confirmed also for some of these genes (*GAPDH*, *ALDOA*, and *NEAT1*) the estradiol dependency in normoxia and the HIF-1α dependency in hypoxia and resistance to fulvestrant (*SI Appendix*, Fig. S4*D*). This list of 31 genes was selected for general and metabolic pathway enrichment analysis. We found enrichment in glycolysis, HIF-1α, and Notch pathways (*SI Appendix*, Fig. S4*E*). The most relevant gene ontology process was the vesicle-transport pathway (*SI Appendix*, Fig. S4*F*).

These data collectively demonstrate that SNAT2 induction, as well other genes, in MCF7 cells is regulated by ERα but becomes predominantly a HIF-1α–dependent gene under hypoxia, due to a potential compensation by HIF-1α when ERα is degraded.

### SNAT2 Is Increased in a Tamoxifen-Resistant Breast Cancer Cell Line in Normoxic Conditions.

SNAT2 expression was analyzed in the tamoxifen-resistant MCF7 cell line (MCF7-TamR) (27). SNAT2 protein was higher in MCF7-TamR cells compared with parental MCF7 cells in normoxia, but not in hypoxia (Fig. 4*F*). Thus, they represent a constitutive SNAT2 model, but with expression within the induced range. When MCF7-TamR cells were grown as a three-dimensional model (spheroids), an increase in cell growth was seen compared with MCF7 parental cells. Interestingly, MCF7-TamR cells were more sensitive to a SNAT2 inhibitor [α-(methylamino)isobutyric acid (MeIAB, 10 mM] compared with the parental cells (maximum volume for MCF7 = 0.56 mm^3^ vs. 0.35 mm^3^ for MCF7-TamR; *P* < 0.001). This corresponds to a 37.5% decrease in cell growth for MCF7-TamR cells after MeIAB treatment versus a 21% decrease in cell growth after MeIAB treatment for parental MCF7 cells (*t* test, *P* < 0.001) (Fig. 4*G*).

### SNAT2 Knockdown Sensitizes Breast Cancer Cells to Antiestrogen Treatment and Decreases Glutamine Consumption, Decreases Mitochondrial Respiration, and Regulates mTORC1 Signaling.

**Fig. 5.**
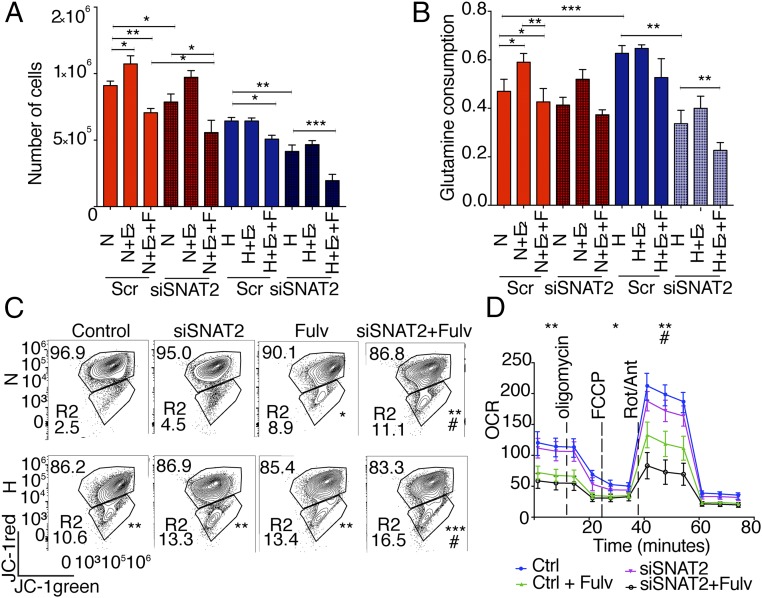
SNAT2 knockdown sensitizes MCF7 cells to fulvestrant treatment in hypoxia and reduces glutamine intake. (*A*) Total of 10^5^ cells were seeded in charcoal-stripped medium and treated with or without E_2_ (10 nM), fulvestrant (10 μM), and *SNAT2* knockdown for 5 d in normoxia (N; red) and hypoxia (H; blue, 1% O_2_) (*n* = 4). Scr, scrambled control. (*B*) Glutamine consumption was calculated as reported from cells grown in the experiment above. (*C*) Determination of mitochondrial membrane potential in MCF7 cells by JC-1 staining in normoxia and hypoxia (0.1% O_2,_ 48 h) with or without *SNAT2* knockdown and with or without fulvestrant (Fulv; 10 μM). Values in the trapeziform regions indicate the proportion of cells compared with the total number of cells. The percentage of depolarized green mitochondria is shown in the R2 box. (*A*–*C*) *P < 0.05, **P < 0.01, ***P < 0.001, ^#^*P* < 0.05 (compared with Fulv treatment) by χ^2^ test compared with controls (*n* = 3). (*D*) Representative plot of O_2_ consumption with or without SNAT2 knockdown with or without Fulv (10 μM) in normoxia (21% O_2_). *P < 0.05, **P < 0.01, ^#^*P* < 0.05 (compared with Fulv treatment); unpaired *t* test (*n* = 3). Ant, antimycin A; Ctrl, control; FCCP, carbonyl cyanide-4-(trifluoromethoxy)phenylhydrazone; OCR, oxygen consumption rate; Rot, rotenone.

To investigate the role of SNAT2 in antiestrogen resistance, we knocked down *SNAT2* with a siRNA pool (double transfection). Cells were treated with E_2_ (10 nM) and/or fulvestrant (10 μg/mL) in normoxia and hypoxia (Fig. 5*A*). Cell growth was followed over 5 d (*SI Appendix*, Fig. S5*A*). Consistent with their ER positivity, MCF7 cells were dependent on E_2_ for their growth in normoxia and sensitive to fulvestrant in normoxia and hypoxia (Fig. 5*A*). SNAT2 knockdown reduced cell proliferation in normoxia, but the inhibitory effect was greater in hypoxia (Fig. 5*A*). A similar experiment was performed with MCF7 parental and MCF7-TamR cells (*SI Appendix*, Fig. S5 *B* and *C*). The latter showed increased growth in both normoxia and hypoxia compared with MCF7 controls. SNAT2 knockdown reversed the growth advantage of TamR cell lines compared with controls in both normoxia and hypoxia. The combination of SNAT2 knockdown and fulvestrant had a greater effect compared with a single treatment. This effect was greater in the TamR cells in both normoxia and hypoxia compared with controls (*SI Appendix*, Fig. S5*C*).

We then investigated if reduced proliferation was linked to reduced glutamine intake. We found that glutamine consumption was increased after E_2_ supplementation in normoxia and decreased after fulvestrant treatment as previously described (16). Glutamine consumption was also increased under hypoxia, but no effects were seen with E_2_ supplementation or fulvestrant treatment in hypoxia. Interestingly, SNAT2 knockdown did not decrease glutamine consumption in normoxia, but a clear effect was seen under hypoxia, with an additive effect when SNAT2 knockdown was combined with fulvestrant (Fig. 5*B*). These data indicate that in MCF7 cells, SNAT2 inhibition sensitizes to hypoxia and antiestrogen treatment and reduces glutamine uptake.

We used flow-cytometry analysis with the cationic dye JC-1 staining to examine the change in mitochondrial membrane potential (ΔΨm) following SNAT2 knockdown in normoxia and hypoxia with or without fulvestrant (Fig. 5*C*). Fulvestrant, hypoxia, and the combination had a significant effect, with 8.9 ± 1.6% (*P* < 0.01), 10.6 ± 1.4%, and 13.4 ± 1.8% (*P* < 0.001), respectively, of the cells showing a loss of mitochondrial membrane potential. When SNAT2 knockdown was performed in hypoxia, 13.3 ± 1.5% of the cells showed a loss of mitochondrial membrane potential (*P* < 0.01; Fig. 5*C*). SNAT2 knockdown after fulvestrant in normoxia (11.1 ± 2.4%) and hypoxia (16.5 ± 2.0%) decreased the mitochondrial membrane potential compared with fulvestrant alone in the same conditions (^#^*P* < 0.05; Fig. 5*C*).

The effect of SNAT2 knockdown on mitochondria respiration was measured by the SeaHorse analyzer. SNAT2 knockdown decreased maximal respiration and ATP production under no-glutamine conditions but not in normal medium or glucose-free medium (*SI Appendix*, Fig. S5*D*). Fulvestrant alone decreased basal and maximal respiration (*P* < 0.01) and ATP production (*P* < 0.05). However, SNAT2 knockdown after fulvestrant administration had a further additive effect on reducing maximal respiration (*P* < 0.01) compared with fulvestrant alone (Fig. 5*D*).

We then assessed the SNAT2 knockdown effect on the mTORC1 pathway. SNAT2 depletion reduced the level of phosphorylated mTOR (p-mTOR) in its downstream targets, p-pS6, and p-p70 (only in hypoxia), compared with their total protein levels (*SI Appendix*, Fig. S5*E*). These results confirmed the ability of SNAT2, similar to other AA transporters, to modulate the mTORC1 signaling cascades (28). Recent research suggested that mTORC1 is also localized at the level of the TGN and can be activated by specific TGN-AA transporters (29). We found that SNAT2 was concentrated on the TGN in MCF7; although most mTOR is localized in the cytoplasm, some colocalization with the TGN was also observed, raising the possibility that it might be associated with SNAT2 in this compartment (*SI Appendix*, Fig. S5*F*).

### SNAT2 Is Induced by AA Deprivation, and Its Overexpression Promotes Resistance to Glutamine Starvation, Hypoxia, and Antiestrogen Treatment In Vitro.

To assess if the shortage of specific AAs can up-regulate SNAT2, we incubated MCF7 cells in the culture medium depleted of several AAs in normoxia and hypoxia. Depletion of glutamine, serine, or glycine (SNAT2 substrates) increased SNAT2 expression in normoxia. Interestingly, when MCF7 cells were incubated under hypoxia, the SNAT2 up-regulation became more profound and independent of single AA deprivation (*SI Appendix*, Fig. S5*G*).

We investigated the growth inhibition after SNAT2 knockdown during metabolic stress. SNAT2 knockdown had an effect on MCF7 growth only under hypoxia in full medium but sensitized MCF7 to glutamine deprivation in two-dimensional growth, particularly under hypoxia (*SI Appendix*, Fig. S5*H*). A small additive effect was also seen when SNAT2 was knocked down in a glucose-free medium.

We then stably overexpressed *SNAT2* by lentiviral vector-mediated transduction in MCF7 cells (SNAT2-o) (*SI Appendix*, Fig. S5*I*). MCF7 SNAT2-o cells were more resistant to fulvestrant in both normoxia and hypoxia (*SI Appendix*, Fig. S5*L*).

Reciprocally, MCF7-SNAT2–overexpressing spheroids grew faster than their parental controls in full and, particularly, glutamine-deprived medium (*SI Appendix*, Fig. S5*M*). These data indicate that in MCF7, SNAT2 inhibition sensitizes to glutamine deprivation and increased expression enhances growth in low-glutamine conditions.

### SNAT2 Overexpression Promotes Resistance to Antiangiogenic and Antiestrogenic Treatment In Vivo.

**Fig. 6.**
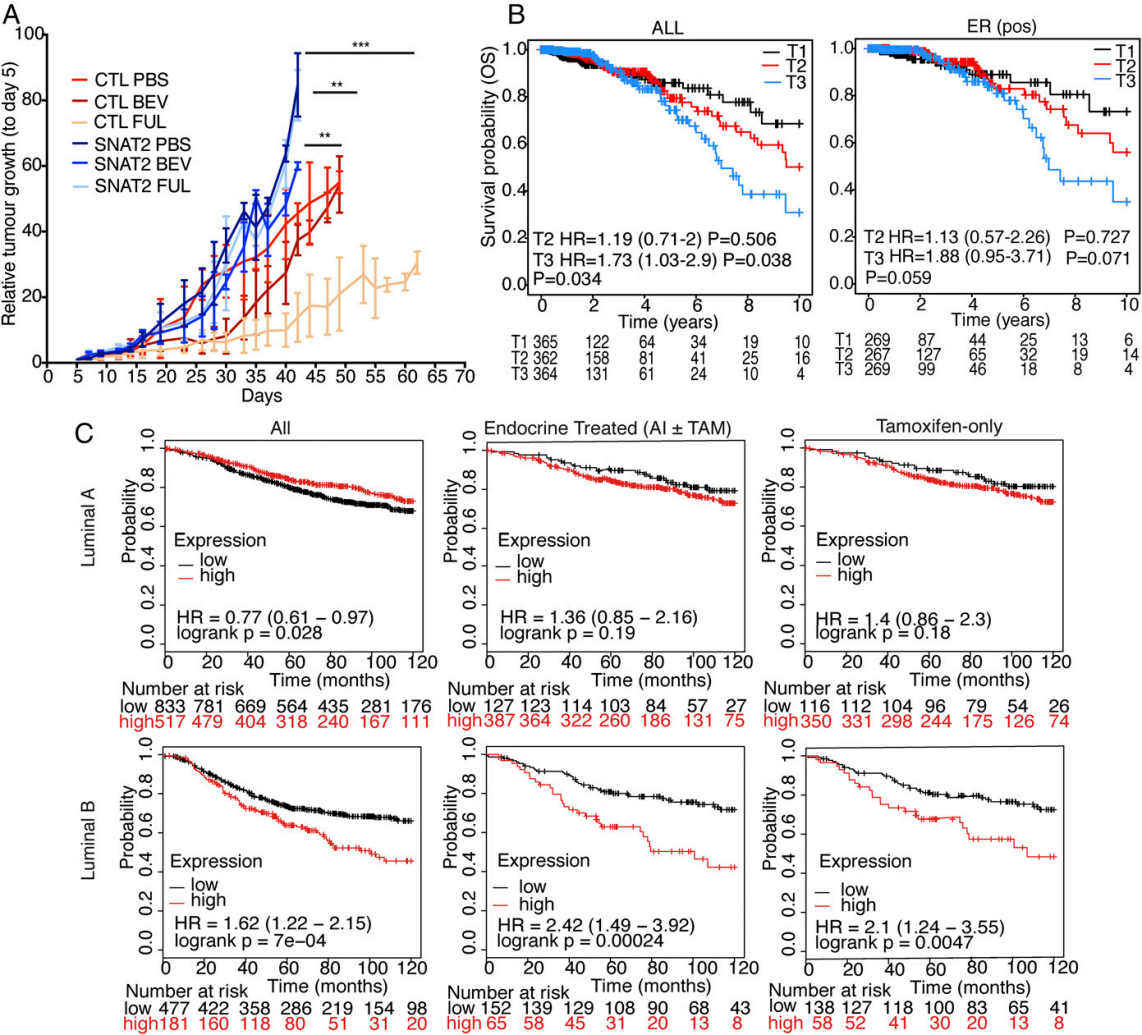
SNAT2 expression is related to poor outcomes in endocrine-treated breast cancer patients. (*A*) Xenograft growth curves of MCF7 parental and SNAT2-o clones ± bevacizumab (BEV) or ± fulvestrant (FULV) treatment. CTL, control. ***P < 0.001, **P < 0.01, *P < 0.05; linear regression followed by Student’s *t* test (*n* = 5). (*B*) Kaplan–Meier plot for patients from the TGCA breast cancer invasive cohort (ALL and ER^+^ cohorts) stratified according to the expression of *SNAT2* mRNA expression (T1, lower tertile; T2, middle tertile; T3, upper tertile). Overall survival (OS) was evaluated. The graph shows that high tumor *SNAT2* levels are associated with increased patient mortality. Subanalysis based on tertile expression showed that the higher SNAT2 mRNA expression (upper tertile) cohort has a lower survival compared with the lower tertile cohort (T3 vs. T1). HR, hazard ratio. (*C*) Kaplan–Meier plots for luminal A and B ER^+^ breast cancer patients (*Left*) and for same subgroups that received endocrine [aromatase inhibitor (AI) or tamoxifen (TAM), *Center*] or tamoxifen only (*Right*). Patients were stratified according to the expression of *SNAT2* mRNA [above (red) versus below (black) third quartile]. Recurrence-free survival was evaluated.

To further assess the role of SNAT2 in resistance to antiestrogen treatment and hypoxia, MCF7 and MCF7-SNAT2-o cells were grown as xenografts with and without bevacizumab or fulvestrant treatment (Fig. 6*A*). MCF7-SNAT2-o xenografts grew faster than the empty vector clone (64.9% the growth rate of the empty vector). Bevacizumab treatment decreased the growth of empty vector tumors in the first phase, and tumors then became resistant. Fulvestrant treatment also reduced the xenograft growth rate (55.4% the growth rate of empty vector control). The SNAT2 expressors grew more rapidly than the controls. The MCF7-SNAT2-o clones treated with bevacizumab grew significantly faster than MCF7 empty vector treated with bevacizumab (11.2% the growth rate of treated empty vector, *P* = 0.038). More strikingly, MCF7-SNAT2-o clones treated with fulvestrant became completely resistant to the antiendocrine treatment (267% the growth rate of treated empty vector, *P* < 0.001). SNAT2-o xenografts, independent of the treatments, had increased proliferation compared with controls as determined by Ki67 staining (*P* < 0.001; *SI Appendix*, Fig. S6*A*).

### SNAT2 Expression Correlates with Tumor Hypoxia and Is Associated with Poorer Recurrence-Free Survival in Endocrine-Treated Breast Cancer Patients.

To evaluate the clinical relevance of SNAT2 in breast cancer, we determined whether tumor *SNAT2* expression levels were correlated with patient prognosis. Using expression data derived from The Cancer Genome Atlas (TGCA) breast invasive cohort (30), we categorized patients according to *SNAT2* tertile expression. Kaplan–Meier analysis of overall mortality (Fig. 6*B*) revealed that patients with high tumor *SNAT2* expression have a significantly poorer outcome compared with patients with low tumor *SNAT2* expression (for trend, *P* = 0.034), with a similar pattern seen in ER^+^ patients (Fig. 6*B*). Moreover, analyzing gene expression data from 2,433 breast cancer patients using the Molecular Taxonomy of Breast Cancer International Consortium (METABRIC)cohort (31), we found that *SNAT2* mRNA abundance significantly correlated with the expression of many genes in our previously reported in vivo hypoxia signature (32), as well as with *HIF-1α*, but not with *HIF-2α* (*EPAS*), and the whole signature itself (*SI Appendix*, Fig. S6*B*). Interestingly *SNAT2* mRNA levels did not correlate with *c-Myc* copy number (*SI Appendix*, Fig. S6*C*). We used additional expression data derived from different breast cancer gene array cohorts, and we tested if the *SNAT2* levels correlated with worse outcomes in ER^+^ patients treated with all adjuvant endocrine treatments (including aromatase inhibitors) or tamoxifen only. High *SNAT2* expression correlated with low recurrence-free survival in patients who received endocrine or tamoxifen treatments (*SI Appendix*, Fig. S6*D*). Moreover, when we looked at the tamoxifen-treated cohort, we found that high *SNAT2* levels correlated with worse outcome in tamoxifen-treated luminal B, but not luminal A, patients (Fig. 6*C*).

## Discussion

Hypoxia is a cause of tumor aggressiveness and resistance to treatments, including endocrine therapy. Although the role of hypoxia and the HIF-1α transcriptional response in promoting tumor progression and metastasis is well established (12), the direct contribution of the HIF family to the regulation of AA transporters has been less studied, apart from LAT1 and glutamate transporters (17). Here, we describe a mechanism by which hypoxia can produce resistance to antiendocrine therapy, by substituting a glutamine transporter for ERα in regulating the AA transporter SNAT2. We found that despite the induction of several AA transporters in hypoxia, SNAT2 expression was key and able to control the growth response to glutamine and antiestrogen treatment in vitro and markedly so in vivo.

Both main SNAT2 isoforms were hypoxia-regulated. Until now, the main isoform has been investigated, while the role of other isoforms has not been studied. Recently, it has been shown that different SLC1A5 isoforms are up-regulated and needed for SLC1A5 activity under AA deprivation (33). Therefore, the role of isoform 2 now requires further investigation.

The maturation of SNAT2 protein and transmembrane localization requires its glycosylation (34), which was maintained in hypoxia. Hypoxia can modulate protein glycosylation, either directly through enhanced expression of enzymes of the hexosamine pathway such as O-GlcNAc transferase (OGT) and O-GlcNAcase or indirectly through the enhancement of glycolysis (35). Moreover, OGT also regulates HIF-1α proteasomal degradation (36). Hypoxia increases the amounts of β1-6GlcNAc–branched *N*-glycans and poly-LacNAc structures (37), and HIF-1α can also directly promote the transcription of genes for fucosyltransferase VII, sialyltransferase ST3Gal-I, and uridine 5′-diphosphate–galactose transporter-1 (38). In this respect, it will be interesting assessing the role of glycosylation induced by hypoxia on proteins relevant to cancer metabolism such as SNAT2.

Accumulating data suggest a significant interplay between hypoxia and estrogen-mediated pathways in breast cancer cells (10, 39). Hypoxia down-regulates ERα in human breast cancer cells via a proteasome pathway (39). We found that both HIF-1α and ERα have binding sites on the same *cis*-regulatory region of the SNAT2 gene, suggesting that SNAT2 transcription can be regulated by either of these two transcription factors, permitting hypoxia-dependent growth under antiestrogen treatment. Of interest was a further set of genes regulated by ERα in normoxia and by HIF-1α in hypoxia independent of ERα. These genes warrant further investigation for roles in hormone independence.

We also found a binding site for HIF-2α in the *cis*-regulatory region of SNAT2. HIF-1α and HIF-2α share common targets, including VEGFA, glucose transporter 1 (GLUT1), and erythropoietin (40). Both HIF transcription factors have different time courses of induction by hypoxia, with HIF-1α peaking earlier, while the activation of HIF-2α occurs later (41). Thus, a potential switch from HIF-1α to HIF-2α has been proposed to maintain the long-term adaptation to hypoxia (42). Although knockdown of HIF-2α in MCF7 cells showed a significant reduction of *SNAT2* mRNA and protein, suggesting also a role of HIF-2α in maintaining SNAT2 induction, we did not find any compensatory induction of HIF-2α in HIF-1α–silenced MCF7 cells in vitro and in vivo. Similarly, a recent systematic analysis of the pan-genomic distributions in cancer cells of the two major isoforms of HIF showed strong support for intrinsically distinct patterns of binding, in which disruption of either HIF gene had very little effect on the binding of the other, with little evidence for cross-compensation at sites that bound both HIF isoforms (43).

Increased glutamine metabolism has been shown to play a role in resistance to medical treatment, including endocrine treatments (44). Luminal (ER^+^) breast cancers are more dependent on oxidative phosphorylation (OXPHOS) than on aerobic glycolysis (45), and the transition from the sensitivity from antiestrogen treatments to a resistant state is also mediated by the promotion of OXPHOS (46). Our data are in agreement with a key role of OXPHOS and glutamine consumption, through SNAT2 up-regulation, in modulating endocrine resistance. Of interest, SNAT2 was also strongly up-regulated by hypoxia in ER^−^ breast cancer cells; thus, its role in triple-negative breast cancer, which is generally more hypoxic and glutamine-dependent (47), deserves further evaluation.

SNAT2 knockdown shifted the mitochondrial membrane potential in both normoxia and hypoxia, suggesting the decrease in cell growth might be mediated by mitochondrial impairment. These data suggest that the dependency on SNAT2 in hypoxia to maintain the TCA cycle cannot be compensated for by other AA transporters, which are not induced by hypoxia in breast cancer cells, confirming recent suggestions that SNAT2 acts as a rescue transporter providing AA intake under stress conditions (48).

SNAT2 was up-regulated in response to withdrawal of its substrates and also leucine and isoleucine. Interestingly serine withdrawal (SNAT2 substrate) did not modify SNAT2 protein expression. ER^+^ breast cancer cell lines are known to be more susceptible to serine depletion, probably due to lack of amplification of phosphoglycerate dehydrogenase, and thus are less addicted to serine metabolism compared with ER^−^ tumors (49, 50). We postulate that other AA transporters, such as ASCT1 (SLC1A4) and ASCT2 (SLC1A5), might be more important for serine uptake in MCF7 cancer cells.

The xenograft models demonstrated a far more profound effect of antiestrogen resistance than the in vitro data. Metabolic modifications are often more profound in vivo because of continuous poor nutrient conditions and hypoxic, acidic, and glucose gradients. Additionally, SNAT2 expression is tightly controlled at the posttranslational level (51). AA starvation results in uncharged transfer RNAs, activating GCN2 and ATF4, which, in turn, will cause translation of the abundant SNAT2 mRNA by a cap-independent mechanism (52). SNAT2 protein is also degraded after ubiquitination (53), and this may occur under nutrient-replete conditions, but it is halted when AAs are depleted. This could explain the more rapid growth rate of SNAT2-overexpressing tumors and could potentially be related to scavenging glutamine.

A well-known downstream effector of ERα is c-Myc (54). It is up-regulated by E_2_, and it plays a critical role in modulating resistance to endocrine therapies, by the unfolded protein response (UPR), in ERα^+^ breast cancer (44, 55). Although SNAT2 is induced by the UPR (56) and Myc was found to selectively bind to the promoter regions of SNAT2 (57), we did not find any correlation between *c-Myc* copy number and *SNAT2* mRNA levels in breast cancer patients.

Finally, our analysis of clinical data available from large RNA expression cohorts suggests that high baseline SNAT2 expression correlates with poor survival in patients with breast cancer and with reduced recurrence-free survival in ER^+^ endocrine-treated breast cancer patients, specifically in the luminal B subtype (ER^+^ subtype). The luminal B subtype has aggressive clinical behavior, with a prognosis similar to that of the HER2-enriched and basal-like groups and a lower sensitivity to endocrine treatment compared with luminal A ER^+^ breast cancer (58). Recent studies showed that high expression of different AA transporters, such as SLC3A2 or SLC7A5, is related to poor outcomes in the luminal B ER^+^ breast cancer subtype (59), suggesting that this subtype might be particularly vulnerable to glutamine depletion. Clinical selection may be possible, as an AA-based positron emission tomography radiotracer, ^18^F-fluciclovine, has recently been investigated in the imaging of breast cancer (60).

As targeting glutamine metabolism is the subject of intensive research due to its potential clinical applications (14), we propose that SNAT2 should be investigated as a predictive biomarker and a potential target for well-defined molecular subtypes (luminal B) of ER^+^ breast cancer patients and as a combination approach to overcome endocrine therapy resistance.

## Materials and Methods

### RNA-Seq and Bioinformatics.

RNA-seq was performed as previously described (24). The sequenced paired-end reads were aligned to human reference genome GRCh38 with transcriptomic information of the genome by Bowtie 2.2.6 and Tophat v2.1. We then estimated the fold changes on the normalized expression level (fragments per kilobase of transcript per million mapped reads) for each gene and its transcriptional isoforms from those mapped reads using Cuffdiff 2.2.1. The means and SDs are calculated over the triplicates of each cell line. ChIP-seq databases were analyzed as previously described (40). For heatmap and hierarchical clustering, supervised exploratory analysis, transcriptomic data were standardized on a z-score scale. K-mean was utilized to determine the optimal number of clusters. Survival analysis of the TGCA and gene arrays dataset (GSE12093, GSE16391, GSE17705, GSE19615, GSE26971, GSE2990, GSE3494, GSE37946, GSE45255, GSE6532, and GSE9195) cohorts was performed in an R statistical environment (v3.3.2) using survival package (v2.41-3).

### Xenograft Studies.

Procedures were carried out after the approval by the institutional review board at the University of Oxford and under a Home Office license. Xenograft experiments were performed in female BALB/c nu/nu (MCF-7 cells) and BALB/c SCID (MDA-MB-231) mice. A total of 2.5 × 10^6^ (MCF-7) cells or 10 × 10^6^ (MDA-MB-231) cells were injected subcutaneously (s.c.) in the lower flank. Mice injected with MCF-7 cells had estrogen (5 μg/mL) added to their drinking water. Once tumors reached 150 mm^3^, the BALB/c nu/nu (MCF-7 cells) and BALB/c SCID (MDA-MB-231) mice received either intraperitoneal (i.p.) bevacizumab (10 mg/kg every 3 d) or vehicle control. For MCF7 ± HIF-1α-o or HIF-1α (^−/−^) xenografts, 50 μL of Matrigel containing 5 × 10^6^ MCF7 ± HIF-1α-o (14) or 10^6^ MCF7 ± HIF-1α-o HIF-1α (^−/−^) was implanted into the mammary fat pad of NSG female mice, aged 5–6 wk, implanted with an estrogen pellet (0.72 mg, 90-d release). When tumors reached 1.44 cm^3^, mice were killed by cervical dislocation (3). For MCF7 parental and MCF7 SNAT2-o experiments, 5 × 10^6^ cells were injected s.c. in the lower flank. Bevacizumab (10 mg/kg) was injected i.p. every 3 d, and fulvestrant (100 mg/kg) was injected from day 15 until death. All xenografts were also implanted with estrogen as seen above. Tumor growth was monitored three times per week measuring the length (L), width (W), and height (H) of each tumor using calipers. Volumes were calculated from the equation 1/6 × π × L × W × H.

### Statistical Analysis.

Statistical analysis was performed and graphs were constructed using GraphPad Prism v6.0. Results are plotted as mean values with SD. Statistical tests and the number of repeats are described in the figure legends. The Student’s *t* test was used for two-sample analyses, and normal distributions were assumed; otherwise, the nonparametric Mann–Whitney test was used. Analysis of variance was used for more than two-sample analyses. Linear regression of log-transformed growth data was used for the xenograft experiments. No samples or experimental repeats were excluded from analyses. No statistical methods were used for the sample size selection of other experiments.

## Supplementary Material

Supplementary File

Supplementary File
