# Supplementary material for: Hypoxia-induced switch in SNAT2/SLC38A2 regulation generates endocrine resistance in breast cancer
Source: Proc Natl Acad Sci U S A. 2019 May 31;116(25):12452–61. doi: 10.1073/pnas.1818521116 (PMC6589752; doi:10.1073/pnas.1818521116)
Supplement: Supplementary File [file pnas.1818521116.sapp.pdf]

Hypoxia-induced switch in SNAT2/SLC38A2 regulation generates endocrine-resistance in breast cancer: Supplementary Material

Methods
Fig. S1. SNAT2 is increased by hypoxia in breast cancer cell lines
Fig. S2. SNAT2 is increased by hypoxia in a wide range of cancer cell lines
Fig. S3. Bevacizumab decreases blood vessels and causes hypoxia and necrosis in breast cancer cell line xenografts
Fig. S4. ERα and HIF-1α occupy the same genomic regions, but they drive SNAT2 expression independently
Fig. S5. SNAT2 overexpression promotes resistance to anti-estrogen treatment and glutamine deprivation
Fig. S6. SNAT2 levels correlate with HIF-1α but not c-Myc expression in breast cancer patients
Table S1. List of 31 genes showing overlapping binding sites for ERα and HIF-1α

www.pnas.org/cgi/doi/10.1073/pnas.1818521116

Methods

Cell culture

Cell lines were available from ATCC (MCF7, T47D, SKBR3, BT474, HCC1806, HCC1954, MDA-MB-231, SUM159, MDA-MB-468, PC3, HCC1954, PC3, DU159), Clare Hall Laboratories (HCT116) or kindly gift of the Prof Ahmed Ahmed laboratory (SKOV3, HEY, OC316). MCF7-TamR cell lines were a kind gift of the Julia Gee laboratory. Cell line authentication was carried out by STR analyses (LGC Standards) 6 months prior to the first submission of the manuscript. Cells were maintained in a humidified incubator at 5% CO_2 and 37°C. For hypoxic exposure, cells were grown in a humidified atmosphere of 0.1% or 1% O_2, 5% CO_2 at 37°C. All cell lines were maintained in DMEM 25mM glucose supplemented with 10% FBS and no antibiotics. All the experiments were performed in 5mM glucose. For MCF7 spheroid culture, aggregation was initiated by plating 1,000–5,000 cells into ultra-low–adherent round-bottom 96-well plates (VWR) with Matrigel and centrifuging these at 2,000 × g for 5 minutes.

Cell Proliferation

Approximately 100,000 cells per well were seeded in triplicate on 6-well tissue culture plates. Each triplicate was treated with 10 µM Fulvestrant (Sigma-Aldrich, I4409-25MG) or treated with SNAT2 siRNA (20nM) and maintained under 21% or 1% O2 for 5 days. Trypsinized cells were counted using the Cellometer Auto T4 Cell Counter (Nexcelom Bioscience). Only the cell population with a size of 13–27 µm was counted. We used α- (Methylamino) isobutyric acid (MeIAB) (M2383, Sigma-Aldrich) at 10mM concentration in MCF7-TAM-r spheroids. Treatment was started on day 3 and renewed every two days. Pictures were taken every 2-3 days.

RNA extraction and RT-PCR

Cells were lysed in Trizol reagent (Invitrogen), and RNA was extracted using ethanol precipitation. RNA quality and quantity were confirmed using the NanoDrop ND-1000 spectrophotometer (Wilmington, USA). Reverse transcription (rt) was performed using the High Capacity cDNA RT kit (Applied Biosystems, Life Technologies, Bleiswijk, Netherlands). QrtPCR was performed using the SensiFAST SYBR No-ROX Kit (Bioline, London, UK). Raw data were analyzed using *Ribosomal Protein L11* (*RPL11*) and *β-actin* as a housekeeping gene. Each PCR reaction was done in triplicate. Primer sequences are reported below.

Oligonucleotides used for RT-PCR

Gene Name	Forward	Reverse
ALDOA	CTGCCAGTATGTGACCGAGA	ACAGGAAGGTGATCCCAGTG
β-ACTIN	CCCAGCACAATGAAGATCAA	CGATCCACACGGAGTACTTG
CA9	CTTGGAAGAAATCGCTGAGG	TGGAAGTAGCGGCTGAAGTC
GAPDH	CCTGCACCACCAACTGCTTA	GGCCATCCACAGTCTTCTGAG
NEAT1	CCAGTTTTCCGAGAACCAAA	ATGCTGATCTGCTGCGTATG
RPL11	CTTTGGCATCCGGAGAAAT	TCCAAGATTTCTTCTGCC TTG
SLC1A1	GCGAGGAAAGGATGCGAGT	GCTGTGTTCTCGAACCAAGACT
SLC1A4	TGTTTGCTCTGGTGTTAGGAGT	CGCCTCGTTGAGGGAATTGAA
SLC1A5	GAGCTGCTTATCCGCTTCTTC	GGGGCGTACCACATGATCC
SLC7A5	CCGTGAACTGCTACAGCGT	CTTCCCGATCTGGACGAAGC
SLC38A1	TGACAGTGCCCGAGGATGATA	AGACATGCCTAAGGAGGTTGTA
SNAT2 tot	ATGAGTTGCCTTTGGTGATCC	ACAGGACACGGAACCTGAAAT
SNAT2 (1)	ATGAAGACAGCAGCAGCTACAG	TCTACATCTGCATAATGGCTGC
SNAT2 (2)	GCCATGCAGGGCAGTTACTA	AATTGCCTTAGCCAGGGGTC

Gene silencing by RNA interference

Reverse transfection of siRNA duplexes (20 nm) was in Optimem (Invitrogen, Waltham, MA, USA) using Lipofectamine RNAiMax (Invitrogen, Carlsbad, CA, USA) according to the manufacturer's instructions. Here reported the siRNA oligonucleotide pools containing three sequences targeting HIF-1α (5′-CAAGCAACTGTCATATATA-3′, 5′-TGCCACCACTGATGAATTA-3′, 5′-TGACTCAGCTATTCACCAA-3′), HIF2α (5′-TAACGACCTGAAGATTGAA-3′, 5′-CAAGCCACTGAGCGCAAAT-3′, 5′-TGAATTCTACCATGCGCTA-3′) were purchased from Eurogentec. Transfections of siRNA duplexes targeting SNAT2 protein (ON-TARGETplus SMARTpool, LQ-007559-01-0002 or a scramble control (ON-TARGETplus SMARTpool) were performed with a double procedure (reverse transfection and subsequent direct transfection after 3 days).

Immunofluorescence

Immunostaining were performed as previously reported (1). Samples were incubated overnight with SNAT2 primary antibodies and then incubated with fluorescently conjugated donkey secondary antibodies and DAPI for 1 h at 37°C followed by a further three 10-min washes in PBST. All

samples were imaged on a Zeiss LSM 780 confocal microscope. Magnification images were obtained using the x40 objective (1.4NA, Oil DIC, Plan-APOCHROMAT). Image J software was used to quantify SNAT2 intensity as previously described (2).

Determination of glutamine consumption

Cells (1×104/well) in a 24 well plate were cultured for 24 hours in medium without phenol red, the medium was collected, and cells were lysed with RIPA buffer (Sigma-Aldrich). Concentrations of glutamine in the medium and in the cell lysate were determined with the Glutamine Detection Assay Kit (Abcam, ab197011). A standard curve was determined for each experiment to calculate the concentration of glutamine in samples as per manufacture guideline. Glutamine levels were calculated and normalized to total protein levels. The glutamine level of normal culture medium was also measured, and the glutamine consumption was calculated as (glutamine in normal medium - glutamine in the medium after culturing cells) and normalized to protein level.

Mitochondrial Permeability Potential

Cells were stained with the cationic dye JC-1 (Thermofisher). At low membrane potentials, JC-1 exists as a monomer and produces a green fluorescence (emission at ≈527 nm) while at high membrane potentials, JC-1 forms aggregates and produces a red fluorescence (emission at ≈590 nm). Briefly, cells were grown as indicated and incubated in 1.25 μm JC1 and analyzed by the LSR-II (BD) flow cytometer. The gating strategy was based on the presence of three cell populations with high (R1) versus low (R2) emission in the PE channel. Data were processed by FlowJo v7.6.4.

Generation of SNAT2 and HIF-1α overexpressing MCF7

Stable MCF7 control or overexpressing the full-length SNAT2 cell lines were generated using viral infections. Briefly, 30 μg of empty vector-containing pLenti6.2-V5 plasmid or full-length SNAT2 cDNA-containing pLenti6.2-V5 plasmid with 15-μg pPAX2 and 5μg of pMDG.2 (Addgene) were transfected into HEK293t packaging cell line using the CaCl2 method. The viral supernatant was recovered and the transduced cells were generated by infection at 5 MOI (multiplicity of infectious units) and selected with 1 μg/mL of Puromycin for 1 week. SNAT2 expression was confirmed by Western blot. The generation of MCF7 HIF-1α overexpressing cells was previously reported (3).

Immunohistochemistry

Immunohistochemistry was carried out as previously described (4). The following primary antibodies were used at overnight incubation: anti-mouse CD31/PECAM (1:100, Clone ER-MP12,

ThermoFisher) mouse anti-CA9 (M75, Absolute antibody, 1:100), rabbit anti-SNAT2 (BMP081, MBL International, 1:100) and Pimonidazole (Hypoxyprobe-1; Chemicon International, 1:100). A standard hematoxylin and eosin protocol was followed to assess the amount of necrosis on xenografts. Slides were incubated with secondary antibody (Dako) for 45 minutes and washed in PBS. 3,3′-Diaminobenzidine (DAB, Dako) was applied to the sections for 7 minutes. The slides were counterstained by immersing in hematoxylin solution (Sigma-Aldrich) and mounted with Aquamount (VWR). Secondary-only control staining was performed routinely. Necrosis was quantified histologically on hematoxylin-stained sections as previously described (4). Expression of CA9 and necrosis was quantified on whole sections by using ImageJ software (US National Institutes of Health, Bethesda, MD, USA). Expression of CD31 was quantified by using 15 random fields of slides at × 100 magnification.

Immunoblotting

Protein concentrations were quantified using a BCA protein assay kit (Thermo Fisher Scientific, Cramlington, UK). Samples containing 30μg of protein were in Laemmli buffer and separated using 8-12% pre-cast SDS- PAGE gels (Bio-Rad, USA). Proteins were transferred to PVDF membranes (Millipore, USA), blocked for 1 hour in transfer buffer (25 mM Tris, pH 7.5, 0.15 M NaCl, 0.05% Tween 20) 5% milk. Primary antibodies were used at 1:1.000 unless otherwise stated. Rabbit anti-SNAT2 (BMP081, MBL International), mouse anti-HIF1α (BD Biosciences, Clone 541, 610958), mouse anti-CA9 (M75, Ab00414-1.1, Absolute); rabbit anti-HIF2α (NB 100-122, Novus Biologicals, 1:500), rabbit anti-ERα (D8H8, 8644, CST), phospho-Ser240/244-ribosomal S6 protein (CST; no. 2215), S6 ribosomal protein (CST; no. 2217), phospho-Ser2448-mTOR (CST; no. 2971), mTOR (CST; no. 2983), phospho-Ser371-p70 S6 kinase (CST; no. 9208), p70 S6 kinase (CST; no. 9202) and actin-HRP (Sigma). SNAT2 was deglycosylated with PNGase F (New England Biolabs, Hitchin, UK; no. P0704) according to the manufacturer's instructions. Appropriate secondary horseradish peroxidase–linked antibodies were used (Dako). Blots were developed on Kodak lm (Sigma, Steinheim, Germany) using ECL plus reagent (GE Healthcare, UK). Image J software was used to quantify bands intensities after β-actin normalization.

Seahorse XF-24 metabolic flux analysis.

Oxygen consumption rates and extracellular acidification rate were measured at 37°C using an XF24 extracellular analyzer (Seahorse Bioscience). MCF7 were plated in 24-well plates for 24 h (25,000 cells per well) in DMEM 5mM glucose, 4mM glutamine with or without SNAT2 knockdown. 24 hours before the experiments fulvestrant (ICI 182,780, 10μM) was administered.

For the other experiment, (50.000 cells per well) were changed to normal 5mM medium or medium with no glutamine or no glucose 24 hours before the experiments. Mitochondrial function was interrogated by the sequential injection of oligomycin A (1.5 μM, which inhibits ATP synthesis), FCCP (0.5 μM, uncoupling agent) and antimycin A (10 μM, complex III inhibitor) in combination with rotenone (2 μM, complex I inhibitor), as per protocol. This allowed for the calculation of ATP-linked O_2 consumption, proton leak, maximal respiratory capacity, reserve capacity, and non-mt respiration. After each experiment, the protein concentrations in each well were measured by Bio-Rad DC™ Protein Assay. Values were normalized to protein concentration after the completion of the XF assay. Three baseline measurements were taken before the sequential injection of mitochondrial inhibitors. Oxygen consumption rate was automatically calculated by Seahorse XF-24 software. Every point represents an average of n = 6 per experiment.

Generation of MCF7 cells lacking functional HIF-1α using CRISPR/Cas9

CRISPR-Cas9 expression constructs were designed and cloned into pSpCas9(BB)-2A-Puro as previously described (5) with minor modifications, as detailed below. The following CRISPR guide sequences (sgRNAs), designed using the MIT CRISPR Design Tool (crispr.mit.edu) were used: HIF-1α_forward: caccgTTCTTTACTTCGCCGAGATC, HIF-1α_reverse: aaacGATCTCGGCGAAGTAAAGAAc. Corresponding guide oligonucleotides were synthesised, mixed, phosphorylated using T4 Polynucleotide Kinase (New England Biolabs), and annealed in a thermocycler using the following programme: 37°C for 30 min, 95°C for 5 min, decrease temperature to 25°C at 0.1°C/min. The empty Cas9 expression plasmid was linearized using BbsI, ligated to annealed oligonucleotides and transformed into DH5α E. coli. Colonies were tested for successful insertion by colony PCR using the forward primer AATTTCTTGGGTAGTTTGCAGTTTT and the reverse guide oligonucleotide, with an expected band at 150bp. MCF7 cells were transfected with the CRISPR-Cas9 expression constructs using FuGENE HD Transfection Reagent (Promega), according to the manufacturer's instructions. The day after transfection 1 μg/mL puromycin was added to the medium for 72 h before cells were seeded at limiting dilutions to obtain monoclonal colonies (500-1000 cells per 15 cm cell culture dish). After 2 weeks colonies (>100 cells) were isolated and expanded until they could be tested for loss of the target protein by western blot.

To verify the sequence of mutated HIF-1α alleles, genomic DNA from HIF-1α-mutant MCF7 cells was extracted using the NucleoSpin Blood kit (Macherey-Nagel), according to the manufacturer's instructions. Exon 2 of the HIF-1α gene, which had been targeted using CRISPR/Cas9, was amplified (forward primer: TTCCATCTCGTGTTTTTCTTGTTGT, reverse primer:

CAAAACATTGCGACCACCTTCT) and PCR products were resolved on a 2% agarose gel. Bands around the expected size (317 bp) were purified and ligated into the pCR4Blunt-TOPO vector using the Zero Blunt TOPO PCR Kit for Sequencing (Thermo Fisher Scientific). Ligation products were transformed into One Shot TOP10 Chemically Competent E. coli cells (Thermo Fisher Scientific). E. coli cells were plated on LB plates containing kanamycin with X-gal (20 μl per plate, 8% w/v in dimethylformamide) and plasmid DNA was amplified and sequenced from 10 blue E. coli colonies (M13 forward primer: TGTAAAACGACGGCCAGT).

References

1. Leiblich A, *et al.* (2012) Bone morphogenetic protein- and mating-dependent secretory cell growth and migration in the Drosophila accessory gland. *Proc Natl Acad Sci U S A* 109(47):19292-19297.
2. Jensen EC (2013) Quantitative analysis of histological staining and fluorescence using ImageJ. *Anat Rec (Hoboken)* 296(3):378-381.
3. Yang J, *et al.* (2015) Estrogen receptor-alpha directly regulates the hypoxia-inducible factor 1 pathway associated with antiestrogen response in breast cancer. *Proc Natl Acad Sci U S A* 112(49):15172-15177.
4. Li JL, *et al.* (2007) Delta-like 4 Notch ligand regulates tumor angiogenesis, improves tumor vascular function, and promotes tumor growth in vivo. *Cancer Res* 67(23):11244-11253.
5. Ran FA, *et al.* (2013) Genome engineering using the CRISPR-Cas9 system. *Nat Protoc* 8(11):2281-2308.

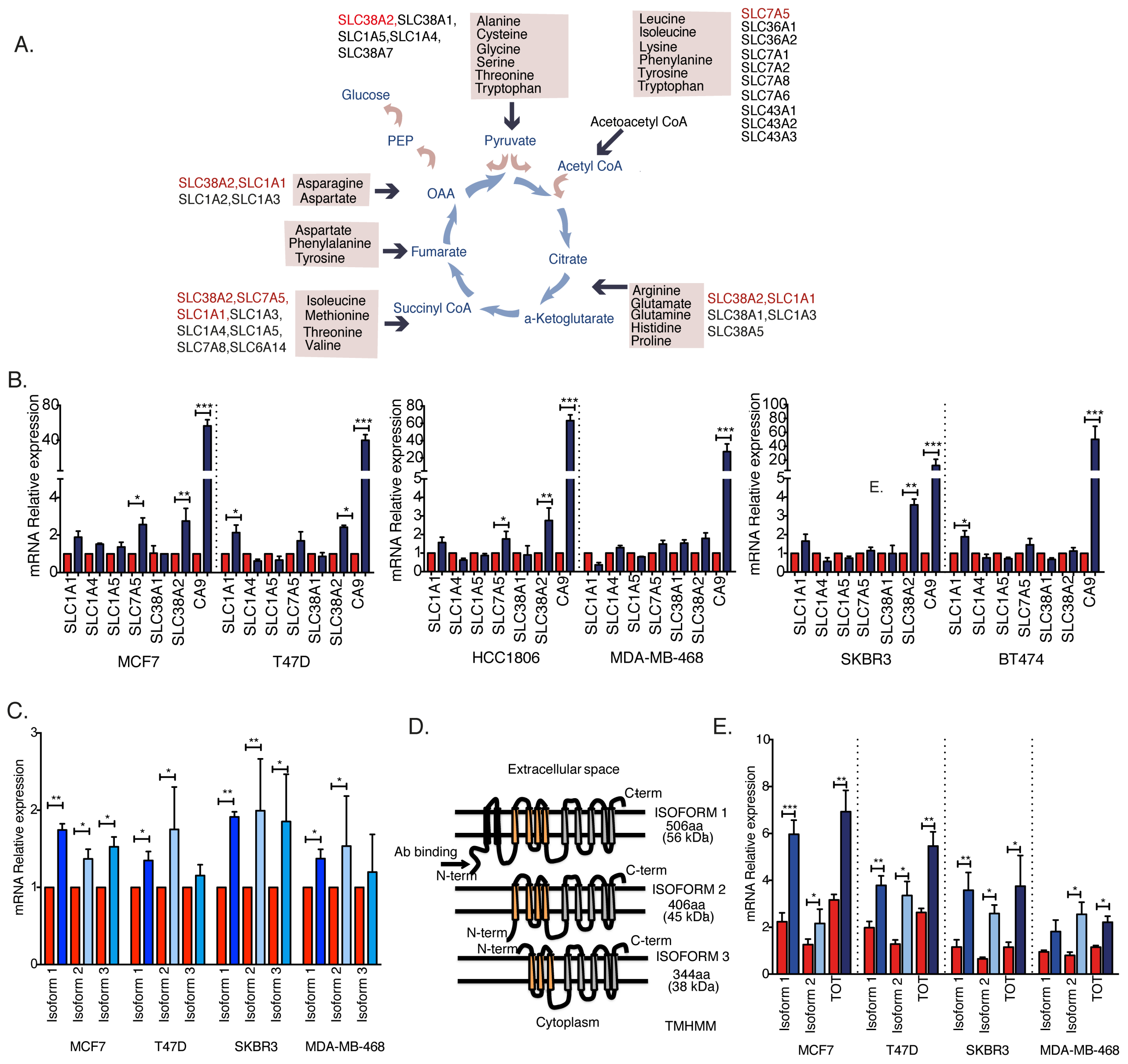

Fig. S1. SNAT2 is increased by hypoxia in breast cancer cell lines

(A) Schematic model showing that the three clustered AA transporters (red) upregulated under hypoxia can potentially fulfill all the AA precursors of TCA cycle metabolites. These three AA transporters can provide ketogenic AA (SLC7A5), gluconeogenic AA (SLC38A2) and glutamate and aspartate (SLC1A1) intake. These AAs can enter the TCA cycle at the level of all the TCA cycle intermediates (Pyruvate; Acetyl-CoA; Citrate; α-ketoglutarate; Succinyl-CoA; Fumarate; OAA, oxaloacetate) throughout reactions of decarboxylation and transamination. **(B)** The relative expression of different AA transporters in hypoxia (0.1% O_2, 48 hours; blue bars) compared to normoxia (red bars) in 6 breast cancer cell lines. **(C)** The expression of the three *SNAT2* isoforms in normoxia (red bars) and hypoxia (0.1% O_2, 48 hours; different blue bars for each isoform), measured by RPKM (Reads per kilobase per million mapped reads) by RNA-sequencing is shown

in 4 breast cancer cell lines. **(D)** Schematic based on TMHMM prediction on the three SNAT2 isoforms identified by RNA-Seq. The binding site for the SNAT2 antibody used for all the experiments recognizing the N-terminus of the protein is shown. **(E)** The relative expression of *SNAT2* isoform 1 and 2 by RT-qPCR is shown in hypoxia (0.1% O_2, 48 hours; different blue bars for isoforms 1 and 2) compared to normoxia (red bars) in 4 breast cancer cell lines. *SNAT2* mRNA was normalized to the mean of *β-actin* and *RPL11*. Error bars, SD. Student t-test ***, $p < 0.001$; **, $p < 0.01$; *, $p < 0.05$; n = 3 for all the experiments

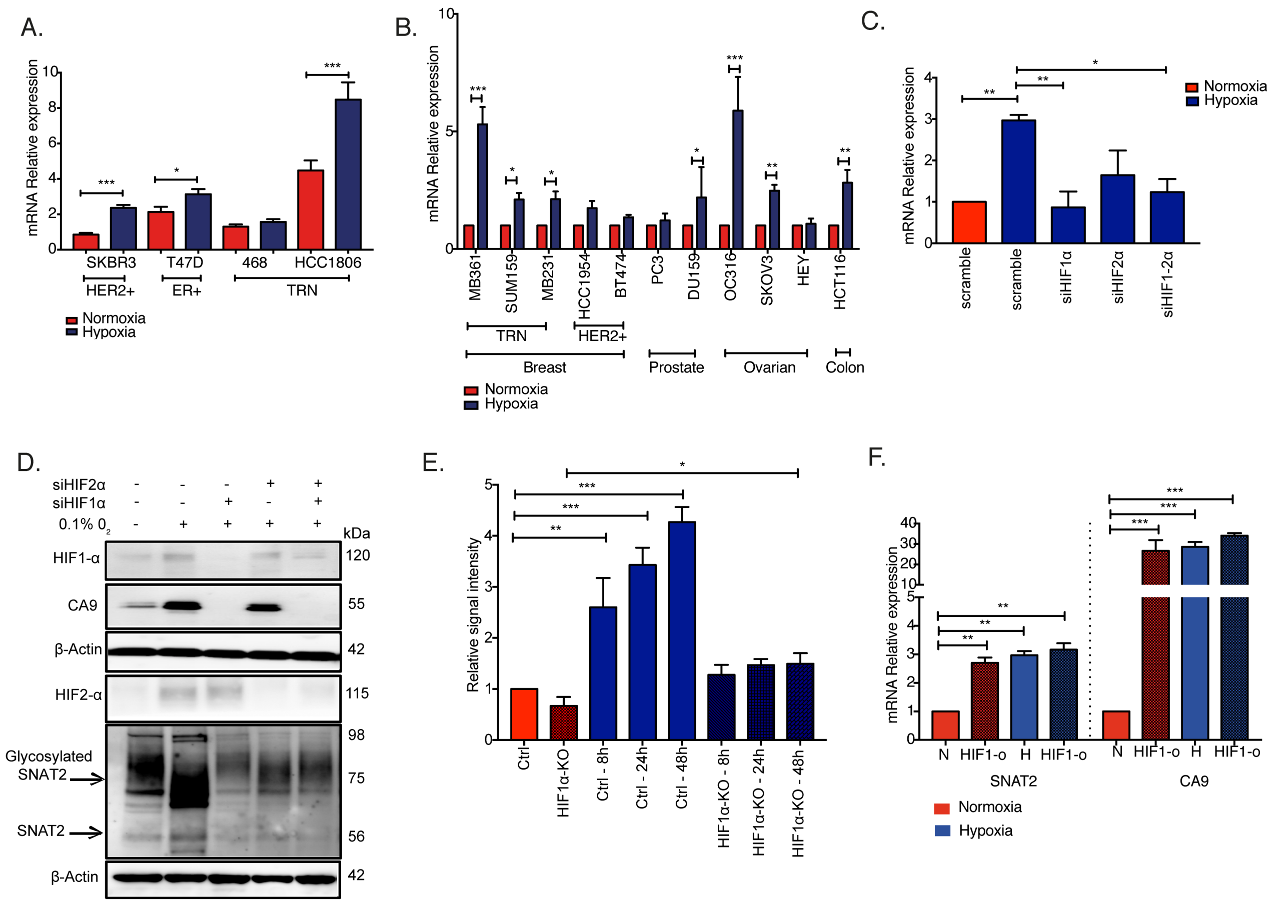

Fig. S2. SNAT2 is increased by hypoxia in a wide range of cancer cell lines

(A) The relative expression of total *SNAT2* mRNA in SKBR3, T47D, MDA-MB-468, and HCC1806 breast cancer cell lines in normoxia and hypoxia. Results are obtained by using the mean of the Ct values of total *SNAT2* transcript after normalization to housekeeping genes *(β-actin* and *RPL11*). **(B)** The relative expression of total *SNAT2* in normoxia (red bars) and hypoxia (0.1% O_2, 48 hours; blue bars) in breast cancer cell lines, prostate cancer cell lines, ovarian cancer cell lines and a colorectal cancer cell line is shown. *SNAT2* mRNA expression was normalized to the mean of *β-actin* and *RPL11*. **(C-D)** MCF7 cells were transfected with scrambled control (scr), or siRNAs against *HIF-1α* (si*HIF1α*), *HIF-2α* (si*HIF2α*) or both and cultured in 21% O_2 or in hypoxia (blue, 0.1% O_2) for 48 hours. mRNAs were analyzed by RT-qPCR and normalized to 21% O_2. *SNAT2* mRNA was normalized to the mean of *β-actin* and *RPL11*. One-way ANOVA test. Error bars, SD. n = 4 **(D)** Immunoblot validation of SNAT2, CA9 (HIF-1α target), HIF-1α and HIF-2α in MCF7 in hypoxia (0.1% O_2, 48 hours) after siRNA knockdown specified. β-actin is shown as a loading control. **(E)** Band densitometry analysis of SNAT2 glycosylated protein expression relative to the loading control of three independent experiments (time course experiment of MCF7 parental and

MCF7-HIF-1α knockout cells in hypoxia, Figure 2D) was performed. One-way ANOVA test. Error bars, SD. **(F)** The relative expression of total *SNAT2* and *CA9* mRNA in MCF7 and MCF7- HIF-1α-o in normoxia and hypoxia is shown. Error bars, SD, Student t-test n = 3. ***, $p < 0.001$; ** $p < 0.01$; * $p < 0.05$; for all the experiments.

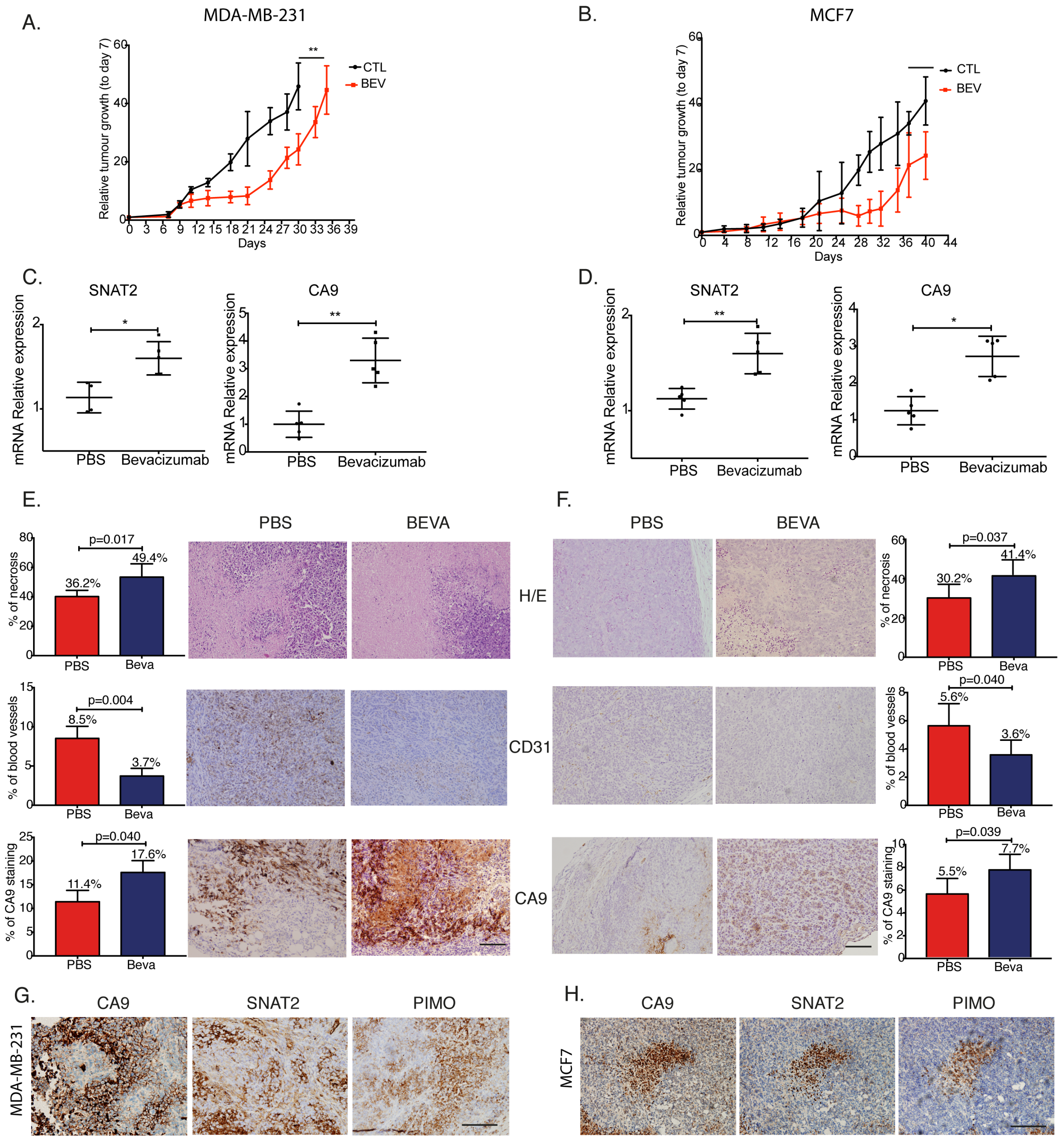

Fig. S3. Bevacizumab decreases blood vessels and causes hypoxia and necrosis in breast cancer cell line xenografts (A-B) Xenograft growth curves of (A) MDA-MB-231 and (B) MCF7 treated with PBS or Bevacizumab. Linear regression followed by Student t-test (**$p<0.01$, *$p<0.05$, n=5). *SNAT2* and *CA9* human mRNAs were increased in (**C-D**) MDA-MB-231 (n=5) and (B) MCF7 (n=5) xenografts treated with bevacizumab. mRNA expression was normalized to the mean of human *β-actin* and *RPL11*. **(E-F)** Representative haematoxylin/eosin (H/E), CD31 (blood vessels) and CA9 (hypoxia) immunostaining in MDA-MB-231 and MCF7 xenografts.

Quantification with ImageJ of the staining for the selected antibodies in control (PBS) and treated (Bevacizumab) xenografts is shown on the lateral side. Scale bars: 100 µm; zoom, ×10. Non-parametric Mann–Whitney test, n=5 per group. Error bars, SD. ** $p < 0.01$, * $p < 0.05$. **(E-F)** Representative SNAT2, CA9 (hypoxia) and Pimonidazole (PIMO, hypoxia) immunostaining in MDA-MB-231 and MCF7 xenografts. Scale bars: 200 µm.

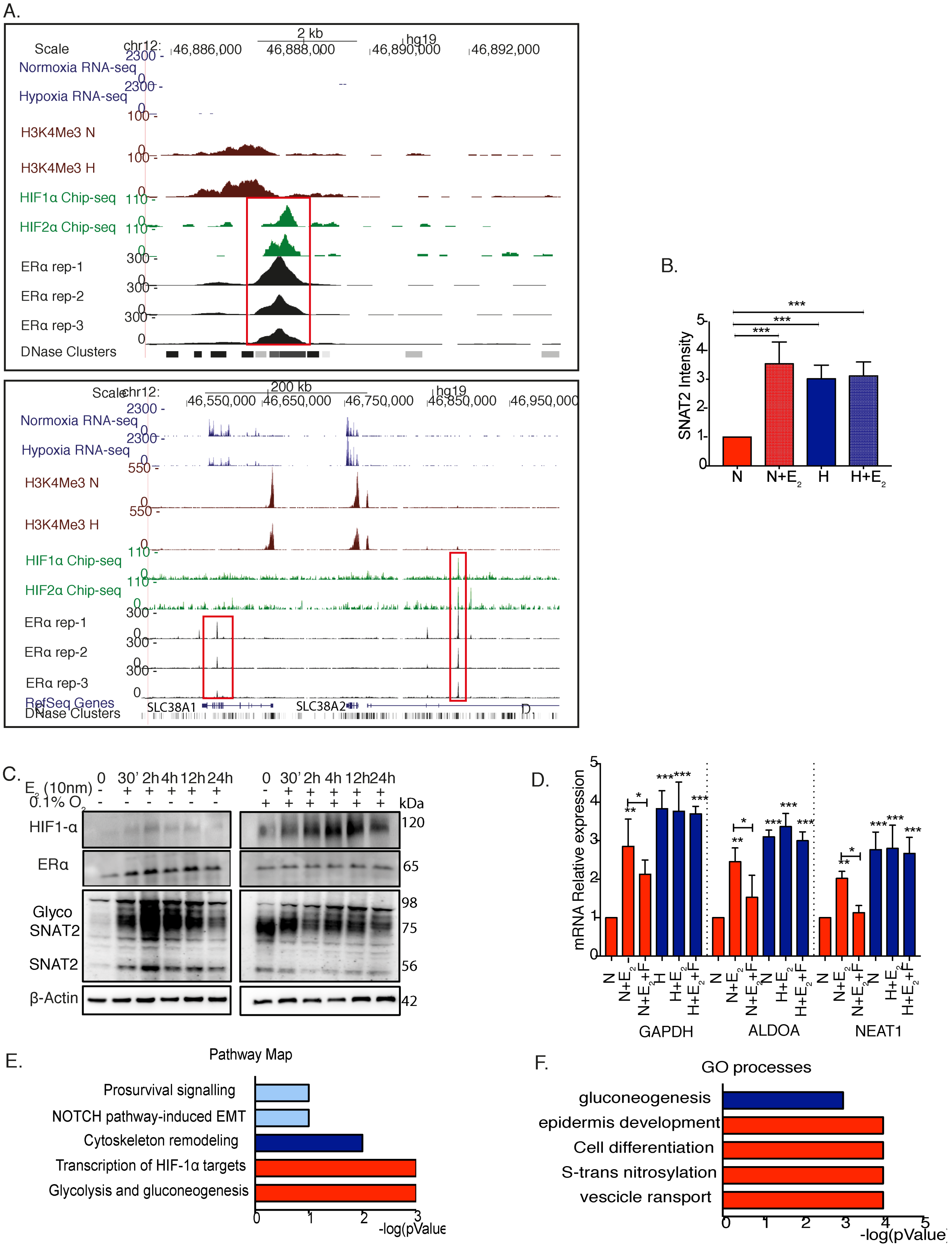

Fig. S4. ERα and HIF-1α occupy the same genomic regions, but they drive SNAT2 expression independently

(A) RNA-seq and ChIP-seq genome-browser view of occupancy (red box) of HIF-1α (top), HIF-2α (middle) and ERα (bottom) at the genomic coordinates of *SLC38A2* on chromosome 12 at high

resolution (upper figure) and lower resolution at the genomic coordinates of *SLC38A1* and *SLC38A2* on chromosome 12 (lower figure). Peaks (red box) represent the areas where transcription factors interact with DNA. **(B)** Quantification with ImageJ of the immunofluorescence intensity for SNAT2 in normoxia (N, red) and hypoxia (H, blue) is presented. Error bars, SD; n = 3.Two-way ANOVA. ***, $p < 0.001$. s*cale bar*, 50 μm. **(C)** MCF-7 cells were treated with 10nM E_2 in 10% charcoal-stripped serum in normoxia (red) and hypoxia (blue, 0.1% O_2) for 30', 2h, 4h, 12h, and 24 hours and subjected to immunoblotting for SNAT2, HIF-1α and ERα. β-actin is shown as a loading control. (**D**) MCF7 cells were grown in charcoal-stripped, phenol-free medium for 3 days and then incubated with or without fulvestrant (ICI 182,780; 10μM) and with or without 10 nM E_2 in normoxia and hypoxia (0.1% O_2) for 48 hours. *GAPDH*, *ALDOA* and *NEAT1* mRNAs were analyzed by RT-qPCR and normalized to 21% O_2. mRNAs were normalized to the mean of *β-actin* and *RPL11*. Error bars, SD. One-way ANOVA. *** $p < 0.001$; **, $p < 0.01$; *, $p < 0.05$ n = 3. **(E)** Pathway map and **(F)** GO processes enriched by MetaCore software of the 31 genes showing overlapping bindings sites for HIF-1α and ERα.

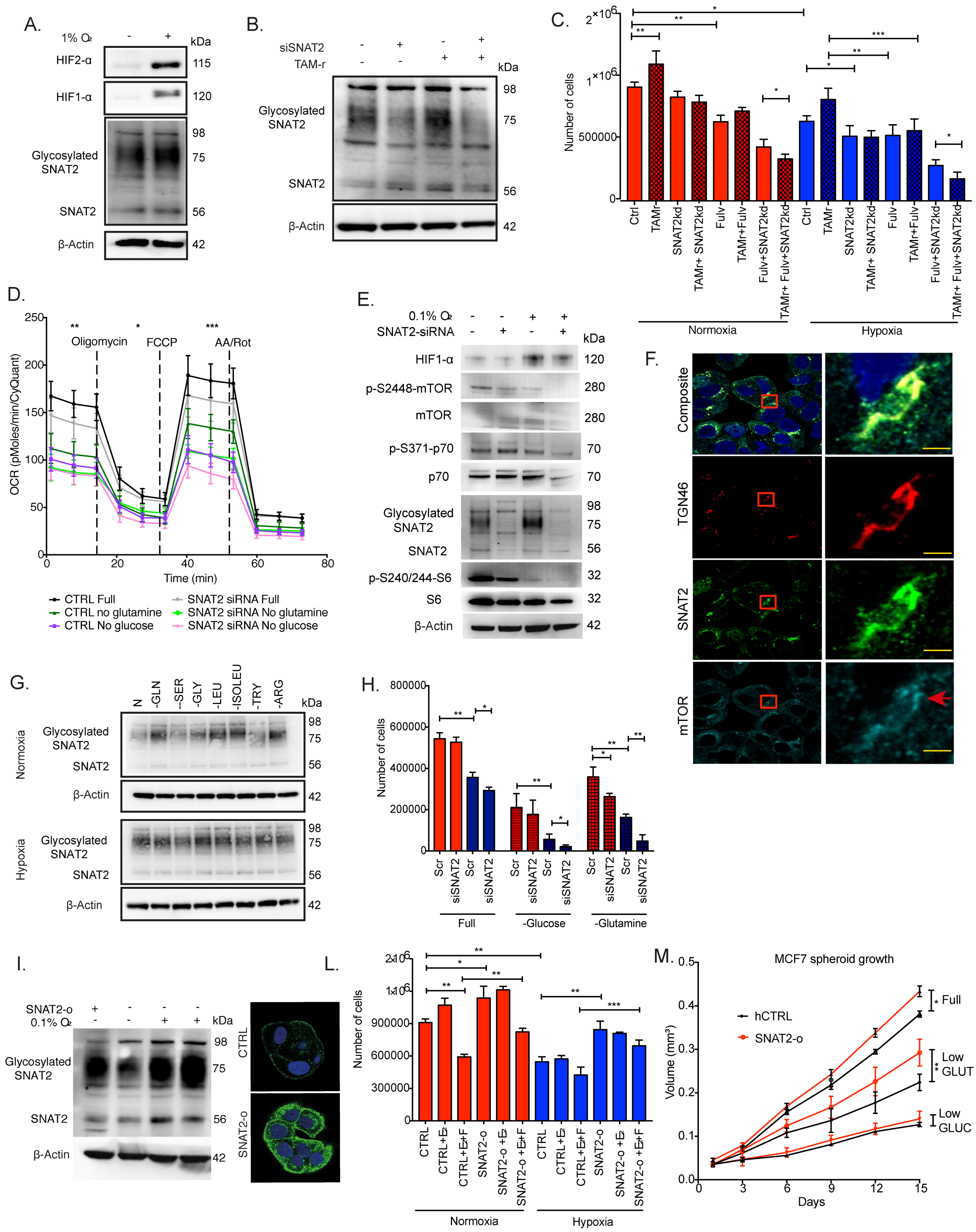

Fig. S5. SNAT2 overexpression promotes resistance to anti-estrogen treatment and glutamine deprivation

(**A**) Representative Western blots of SNAT2, HIF-1α, and HIF-2α in MCF7 cell line in normoxia and hypoxia after 5 days in hypoxia (1% O_2). β-actin is shown as a loading control; n=3 (**B**) Representative Western blots of SNAT2 knockdown (siSNAT2) in MCF7 parental and

MCF/TAM-r cell line in normoxia. β-actin is shown as a loading control; n=3 **(C)** 10^5 MCF7 controls (Ctrl) and MCF/TAM-r cells were seeded in charcoal-stripped medium and treated with or without fulvestrant (ICI 182,780; 10uM) ± *SNAT2* knockdown for 5 days in normoxia (N, red, 21% O_2) and hypoxia (H, blue, 1% O_2); n=3. *** $p < 0.001$; **, $p < 0.01$; *, $p < 0.05$. One-way ANOVA **D)** A representative plot of oxygen consumption measured in MCF7 cells with or without SNAT2 knockdown in normal, glutamine-free or glucose free-medium using the Seahorse Bioscience XF Analyser in normoxia (21% O_2). Lines indicate the time when the oligomycin, the mitochondrial uncoupler FCCP, and AA/rotenone were added. **(E)** Representative Western blots of SNAT2 knockdown in MCF7 cell line in normoxia and hypoxia. β-actin is shown as a loading control **(F)** SNAT2 and mTOR are partially colocalized on the Trans-Golgi network (TGN) in MCF7 cells. mTOR (blue) is mainly located in the cytoplasm (probably late endosomes and lysosomes) (blue) but some mTOR staining also overlaps with the TGN (red) and SNAT2 (green). DAPI marks the nucleus. Scale bars are 5 µm. **(G)** MCF7 cells were incubated in tissue culture medium depleted of the indicated amino acids in normoxia or hypoxia for 24 hours. Immunoblot analysis of SNAT2 is shown. β-actin is shown as a loading control; n=3 **(H)**10^5 MCF7 cells were treated with scramble (scr) or with *SNAT2* knockdown (siSNAT2) and seeded in normal, glutamine-free or glucose-free medium for 3 days in normoxia (N, red, 21% O_2) and hypoxia (H, blue, 1% O_2); n=3. One-way ANOVA **(I)** Representative Western blots of SNAT2 overexpression and SNAT2 hypoxic levels. β-actin is shown as a loading control. Confocal imaging of SNAT2 (green) overexpression in MCF7 (MCF7-SNAT2-o) cell line versus parental control cells is also shown. DAPI (blue) Scale bars are 10 µm **(L)** 10^5 MCF7 parental (CTRL) and MCF7-SNAT2-o cells were seeded in charcoal-stripped medium and treated with or without E_2 (10nM) and fulvestrant (F, ICI 182,780; 10uM) for 5 days in normoxia (N, red, 21% O_2) and hypoxia (H, blue, 1% O_2); n=4. One-way ANOVA **(M)** Representative graph of the effect of SNAT2 overexpression (red) versus control cells (black) in MCF7 spheroid growth in different medium (normal DMEM (5mM), low glucose (1mM), low glutamine (1mM)). Two-way ANOVA. Error bars, SD. ***, $P < 0.001$; **, $P < 0.01$; *, $P < 0.05$ for all the experiments.

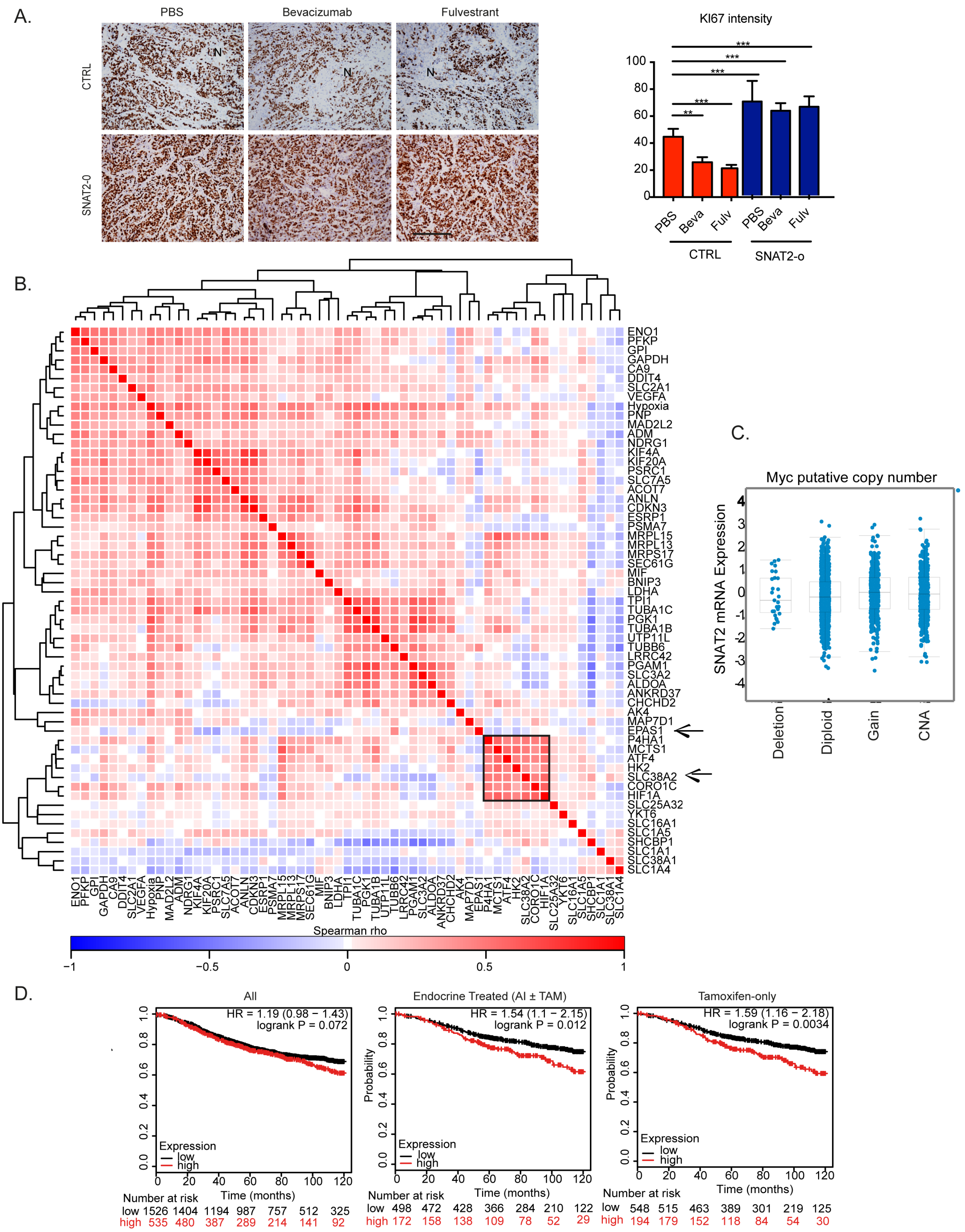

Fig. S6. SNAT2 levels correlate with HIF-1α but not c-Myc expression in breast cancer patients

(A) Analysis of Ki67 expression by immunohistochemistry with representative images MCF7 parental or SNAT2-o xenografts treated with Bevacizumab or Fulvestrant and a bar chart of the

scoring (right). Ki67 expression staining is dark brown. N denotes areas of necrosis. Scale bars represent 100µm. One-way ANOVA (***p<0.001, **p<0.01, *p<0.05, n=5). **(B)** Correlation heatmap of *SNAT2* (*SLC38A2*, arrow) and other genes part of the hypoxia signature, AA transporters, *HIF-1α*, and *ATF4*. *SNAT2 (SLC38A2)* co-expresses (black box) with *HIF-1α* and other genes (black box) previously identified as part of the hypoxia signature but not *HIF-2α (EPAS)*(arrow) in breast cancer patients. Each square represents the Pearson correlation (r) between a pair of genes, calculated using microarray expression data from the Metabric cohort. Red colors indicate a high gene-gene correlation while the opposite is seen for the blue. **(C)** *Myc* copy number and *SNAT2* mRNA expression do not correlate in the Metabric breast cancer cohort. **(D)** Kaplan–Meier plot for ER+ breast cancer patients from different gene-array studies stratified according to the expression of *SNAT2* mRNA (above [red] versus below [black] third quartile) (left panel). High tumor SNAT2 levels are associated with decreased recurrence-free survival only in patients who received endocrine [aromatase inhibitor (AI) or tamoxifen (TAM), middle panel] or tamoxifen (right panel) as part of their treatment for breast cancer.

Table S1: List of 31 genes showing overlapping binding sites for ERα and HIF-1α

Gene	Name	Chr	Start	End
ALDOA	aldolase A, fructose-bisphosphate	chr16	30064411	30081778
ALOXE3	arachidonate lipoxygenase 3	chr17	7999218	8022365
C11orf49	chromosome 11 open reading frame 49	chr11	46958240	47185936
C14ORF43	Homo sapiens ELM2 and Myb/SANT-like domain containing 1 (ELMSAN1)	chr 14	74181825	74253896
CCNL1	cyclin L1	chr3	156864297	156878549
CLIC6	chloride intracellular channel 6	chr21	36041688	36090525
CRKL	v-crk avian sarcoma virus CT10 oncogene homolog-like	chr22	21271714	21308037
CUX1	Homo sapiens cut-like homeobox 1	chr7	101460882	101901513
DAPK2	death-associated protein kinase 2	chr15	64199235	64364232
DNASE1	deoxyribonuclease I	chr16	3661729	3730144
DSP	desmoplakin [Source:HGNC Symbol;Acc:3052]	chr6	7541808	7586950
NEAT1	Homo sapiens nuclear paraspeckle assembly transcript 1, non-coding RNA	chr11	65190269	65212028
FLJ16641	Homo sapiens leucine, glutamate and lysine rich 1 (LEKR1)	chr3	156544148	156643045
GADD45B	growth arrest and DNA-damage-inducible, beta	chr19	2476120	2478257
GAPDH	glyceraldehyde-3-phosphate dehydrogenase	chr12	6643093	6647537
HEY1	hes-related family bHLH transcription factor with YRPW motif 1	chr8	80676245	80680098
ILVBL	ilvB (bacterial acetolactate synthase)-like	chr19	15225795	15236596
FAM174b	Homo sapiens family with sequence similarity 174, member B	chr15	93160679	93199031
MDGA2	Homo sapiens MAM domain containing glycosylphosphatidylinositol anchor 2	chr14	47340143	48095728
N4BP3	NEDD4 binding protein 3	chr5	177540444	177553088
PC	pyruvate carboxylase	chr11	66615704	66725847
QSOX1	Homo sapiens quiescin Q6 sulfhydryl oxidase 1	chr1	180123968	180167169
RNF19	Homo sapiens ring finger protein 19A, E3 ubiquitin protein ligase	chr8	101269288	101315487
S100A10	S100 calcium binding protein A10	chr1	151955391	151966866
SCARB1	scavenger receptor class B, member 1	chr12	125261402	125367214
SLC38A2	solute carrier family 38, member 2	chr12	46751972	46766650
ST8SIA6	ST8 alpha-N-acetyl-neuraminide alpha-2,8-sialyltransferase 6	chr10	17360382	17496329
TMPRSS3	trefoil factor 1	chr21	43782391	43786703
TFRC	transferrin receptor	chr3	195754054	195809060
ANO6	Homo sapiens anoctamin 6 (ANO6)	chr12	45609770	45826134
TMEM88	Homo sapiens transmembrane protein 88	chr17	7855065	7856099
